# Leaderless Transcripts and Small Proteins Are Common Features of the Mycobacterial Translational Landscape

**DOI:** 10.1371/journal.pgen.1005641

**Published:** 2015-11-04

**Authors:** Scarlet S. Shell, Jing Wang, Pascal Lapierre, Mushtaq Mir, Michael R. Chase, Margaret M. Pyle, Richa Gawande, Rushdy Ahmad, David A. Sarracino, Thomas R. Ioerger, Sarah M. Fortune, Keith M. Derbyshire, Joseph T. Wade, Todd A. Gray

**Affiliations:** 1 Department of Immunology and Infectious Diseases, Harvard School of Public Health, Boston, Massachusetts, United States of America; 2 Wadsworth Center, New York State Department of Health, Albany, New York, United States of America; 3 Broad Institute of Harvard and MIT, Cambridge, Massachusetts, United States of America; 4 ThermoFisher Scientific BRIMS Center, Cambridge, Massachusetts, United States of America; 5 Department of Computer Science, Texas A&M University, College Station, Texas, United States of America; University of Geneva Medical School, SWITZERLAND

## Abstract

RNA-seq technologies have provided significant insight into the transcription networks of mycobacteria. However, such studies provide no definitive information on the translational landscape. Here, we use a combination of high-throughput transcriptome and proteome-profiling approaches to more rigorously understand protein expression in two mycobacterial species. RNA-seq and ribosome profiling in *Mycobacterium smegmatis*, and transcription start site (TSS) mapping and N-terminal peptide mass spectrometry in *Mycobacterium tuberculosis*, provide complementary, empirical datasets to examine the congruence of transcription and translation in the *Mycobacterium* genus. We find that nearly one-quarter of mycobacterial transcripts are leaderless, lacking a 5’ untranslated region (UTR) and Shine-Dalgarno ribosome-binding site. Our data indicate that leaderless translation is a major feature of mycobacterial genomes and is comparably robust to leadered initiation. Using translational reporters to systematically probe the *cis*-sequence requirements of leaderless translation initiation in mycobacteria, we find that an ATG or GTG at the mRNA 5’ end is both necessary and sufficient. This criterion, together with our ribosome occupancy data, suggests that mycobacteria encode hundreds of small, unannotated proteins at the 5’ ends of transcripts. The conservation of small proteins in both mycobacterial species tested suggests that some play important roles in mycobacterial physiology. Our translational-reporter system further indicates that mycobacterial leadered translation initiation requires a Shine Dalgarno site in the 5’ UTR and that ATG, GTG, TTG, and ATT codons can robustly initiate translation. Our combined approaches provide the first comprehensive view of mycobacterial gene structures and their non-canonical mechanisms of protein expression.

## Author Summary

The current paradigm for bacterial translation is based on an mRNA that includes an untranslated leader sequence containing the ribosome-binding site upstream of the initiation codon. We applied genome-scale approaches to map the protein-coding regions in the genomes of *Mycobacterium smegmatis* and *Mycobacterium tuberculosis*. We found that nearly one-quarter of mycobacterial transcripts are leaderless in mycobacterial species, thus indicating that ribosomes must recognize these mRNAs by a novel mechanism and suggesting that there are alternative modes of bacterial translation beyond the *Escherichia coli* paradigm. Our translational profiling showed that many mycobacterial proteins are mis-annotated, and also found many new genes encoding small proteins that had been previously overlooked, which are likely to play novel roles in diverse cellular processes. We also developed a new reporter system that provides mechanistic insights into translation initiation through deep sequencing. Our data show that leaderless translation is a robust process that is conserved in mycobacteria, that leaderless translation only requires that the mRNA begin with a start codon, and predict that mycobacteria encode hundreds of small proteins. This work will help us understand gene structure, genome organization and protein expression in bacteria, and how the translational machinery differs in different organisms.

## Introduction

The mechanism of bacterial translation initiation has been the subject of intensive study in the model organism, *Escherichia coli*. Canonical translation initiation in bacteria is a multistep process that begins with the binding of a small (30S) ribosomal subunit to a Shine-Dalgarno element in the 5’ UTR of an mRNA. The Shine-Dalgarno sequence is generally centered 8–9 nt upstream of the start codon and interacts with a complementary sequence in the 16S rRNA of the 30S ribosomal subunit to help position this ribosomal subunit [1]. A complex, comprising the mRNA-bound 30S subunit, fMet-tRNA, and initiation factors, recruits the large (50S) ribosomal subunit, forming a complete 70S ribosome for initiating translation of the open reading frame (ORF). Although almost all studies of bacterial translation initiation have focused on mRNAs that contain a Shine-Dalgarno, non-canonical mechanisms of translation initiation have also been described, including re-initiation and leaderless translation.

Translation of genes embedded in polycistronic mRNAs can be coupled to translation of the respective upstream gene in a process known as re-initiation, if the downstream start and upstream stop codons are positioned close to one another. Once translation of the upstream gene has terminated, the ribosome scans until it finds a suitable start codon further downstream [2]. Low levels of ribosome recycling factor (RRF) impair post-termination disassembly, allowing intact ribosomes to re-initiate translation [3]. Closely spaced or overlapping stop/start codons lead to the most efficient coupling [4]. The precise mechanism of re-initiation is unclear.

“Leaderless” transcripts lack a 5’ UTR and Shine-Dalgarno sequence. In such cases, 70S ribosomes must bind directly to the first nucleotide of the mRNAs to initiate translation. Few leaderless genes have been identified and analyzed in *E*. *coli*, and those that have are mostly of mobile DNA origin, including from λ and P2 phage, and from Tn1721 [5–8]. More recently, the expansion of genome sequence information, gene expression data, and computational analyses into bacterial species other than *E*. *coli*, suggest that leaderless gene structures may not be so unusual [9–17]. Leaderless genes also appear to be more common in some archaeal species [18–22] and in mitochondria [23–25]. These isolated reports hint at a very broad distribution of leaderless genes, suggesting that it is an ancient—and possibly the original—mode of translation initiation [18, 21, 26]. Paradoxically, almost all mechanistic studies of bacterial leaderless translation have used *E*. *coli* as a model system, despite the relative paucity of leaderless genes in this species. It is likely that fundamental differences in translation underlie robust leaderless translation in those species with pervasive leaderless gene structures.

Leaderless translation is poorly understood, compared with canonical leadered translation. Early studies established the absolute requirement for a 5’ ATG (AUG in the mRNA) for translation [27–29]. Leaderless translation is less robust in *E*. *coli* than leadered and early studies focused on identifying factors that contributed to this difference. Leaderless transcripts are likely recognized by 70S ribosomes, rather than the 30S subunit [30–32]. 70S binding is stimulated by initiation-factor 2 [33], whereas initiation-factor 3 inhibits leaderless translation likely by destabilizing codon-anticodon interactions [33, 34], an effect enhanced by the S1 protein [32, 35–37]. More recent studies suggest that at least some leaderless translation is mediated by a distinct sub-population of 70S ribosomes that have been modified by the stress-induced endoribonuclease, MazF [37, 38]. MazF cleaves the 3’ end of the 16S rRNA, thereby removing the anti-Shine-Dalgarno sequence. Hence, these so-called “stress-ribosomes” fail to initiate translation of canonical leadered mRNAs but their translation initiation of leaderless transcripts is unimpaired. Thus, based on *E*. *coli* studies, leadered and leaderless translation processes are functionally distinct.

In contrast to bacteria, eukaryotes utilize ribosome-scanning translation initiation mechanisms. Small open reading frames are frequently found in the 5’ UTRs of eukaryotic genes, and these upstream ORFs (uORFs) can attenuate translation of the annotated downstream ORF [39–41]. Less clear is whether similar upstream ORFs meaningfully regulate expression of the downstream proteins in prokaryotes. In bacteria, short ORFs have been identified upstream of some genes. However, this phenomenon has been described far less frequently than uORFs in eukaryotes [42]. Independent of putative roles in *cis*-regulating downstream ORFs, short ORFs may encode small proteins with *trans* activities. Regulatory functions have been attributed to encoded small proteins themselves [43] in response to environmental cues such as amino acid availability [44], global translation levels [45], and ATP abundance [46].

The genus *Mycobacterium* includes major pathogens as well as non-pathogens. There have been very few studies of translation initiation mechanisms in mycobacteria, although a recent study inferred widespread leaderless translation in *Mycobacterium tuberculosis* by mapping transcription start sites (TSS) onto an annotated reference genome[9]. Here, we use RNA-seq coupled with ribosome profiling to map RNAs and translated RNAs, respectively, in the model mycobacterial species, *Mycobacterium smegmatis*. In parallel, we use TSS mapping coupled with mass spectrometry detection of protein N-termini to map the sites of initiation of the transcriptome and proteome of *M*. *tuberculosis*. These complementary approaches in related species reveal widespread non-canonical translation initiation, including leaderless translation and the use of alternative start codons. Lastly, we developed a next-generation sequencing-based translational reporter system, which allows a controlled assessment of the requirements of translation initiation in mycobacteria. This assay demonstrated that the requirements for leaderless and leadered translation are distinct, and supports the use of alternative start codons for leadered translation. Thus, our collective data provide the first systems-level description of key translational parameters in mycobacteria, a genus that exhibits significant endogenous leaderless translation.

## Results

### RNA-seq and ribosome profiling analyses of *M*. *smegmatis* indicate widespread leaderless translation

The initial report of leaderless translation in *M*. *tuberculosis* relied on annotation pipeline predictions [9], which in turn are predicated on rules derived from canonical leadered translation systems. To generate a dataset that did not inherently rely on annotation predictions, we sought to empirically determine the genomic placement of active transcripts as well as their translated ORFs. The coordinate application of RNA-seq and ribosome profiling allowed simultaneous assessment of the relative levels of all transcripts and their translated regions genome-wide in *M*. *smegmatis* without reliance on annotations. As each genome-wide approach has strengths and weaknesses, combining independent criteria or datasets provides higher confidence results. In this study, we used standard growth conditions including rich media and harvesting RNA from cells at mid-exponential phase to profile those genes most relevant to common experimental conditions. Mapped read depth tended to be greatest near the 5’ ends of the genes, facilitating our search for TSSs and translation initiation sites as the leading edge of stacked sequence reads from RNA-seq and ribosome profiling, respectively.

**Fig 1:**
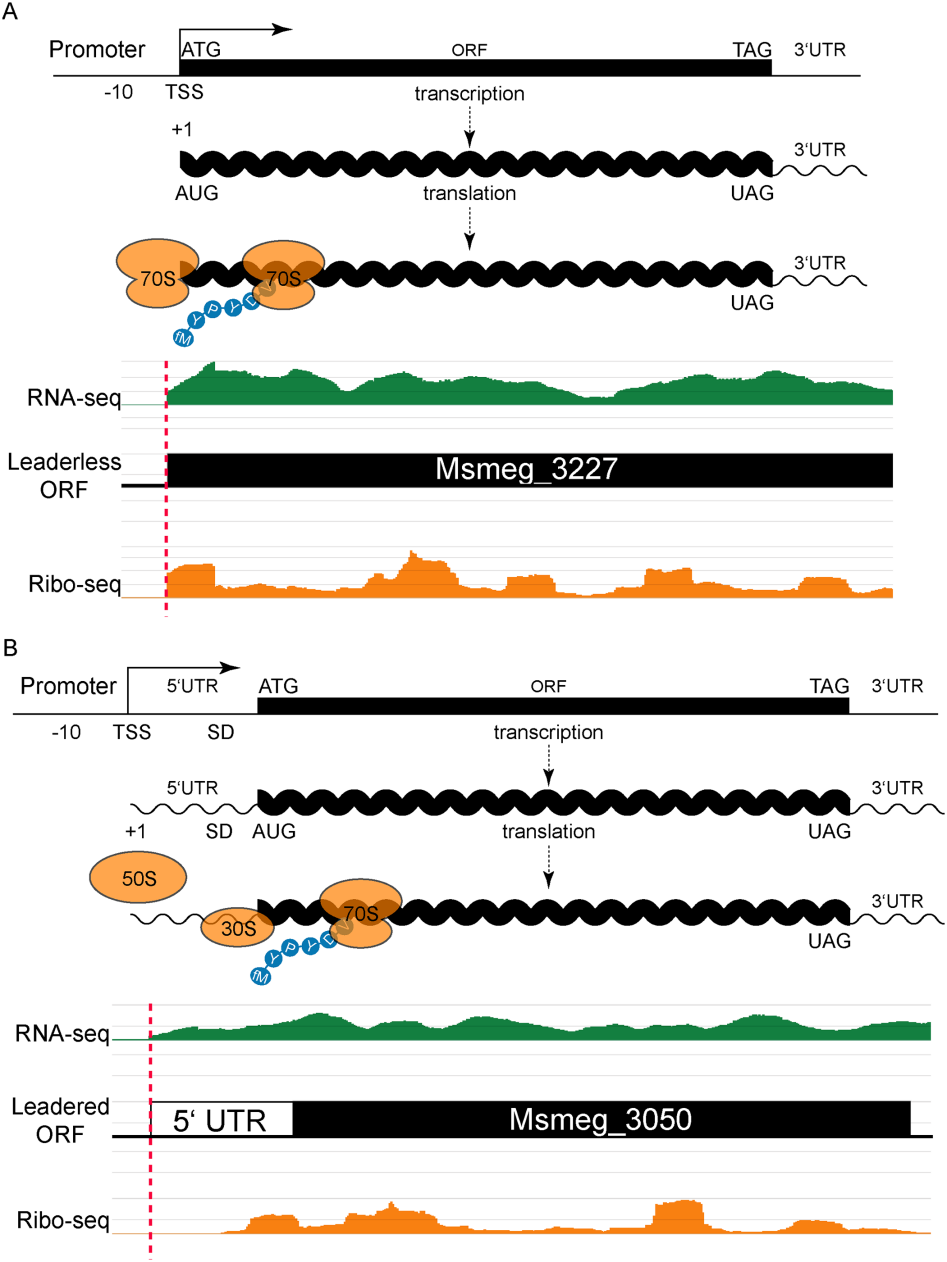
Leaderless and leadered genes produce distinct RNA-seq and ribosome profiling 5’ boundaries. (A) The transcription start site (TSS) and translation initiation site are the same in leaderless genes. No 5’ UTR and no Shine-Dalgarno (SD) sequence imply that an assembled 70S ribosome engages the 5’ terminal initiation codon directly, followed by elongation to translate the ORF. Individual sequence reads from RNA-seq (green) and ribosome profiling (Ribo-seq, orange) analyses were mapped to the genome, and the abundance of the individual reads is indicated by the height of the peaks. In leaderless translation RNA-seq and ribosome profiling have coincident 5’ boundaries. The 5’ triplet is nearly always ATG or GTG and, in the typical example shown, corresponds to the predicted N-terminus of the annotated ORF. (B) Traditional gene structures generate nested ribosome profiling profiles, with a 5’ UTR that includes an SD ribosome-binding site upstream of the initiating methionine codon (ATG). The 30S and 50S ribosomal subunits assemble at the SD to form a complete 70S ribosome that begins translation at the adjacent AUG with an N-terminal formylated methionine (fM) amino acid residue. RNA-seq reads (green) indicate positive-strand transcription at this locus, and upstream of an annotated ORF. Mapped ribosome profiling reads (orange) begin downstream of the onset of RNA-seq reads, and ~17–35 nt upstream of the initiation codon of the annotated ORF. In both examples, JCVI correctly predicted the respective ORFs (black).

Focusing on the upstream boundaries of regions of sequencing signal in either the total RNA or ribosome profiling datasets, we identified putative TSSs and translation initiation sites for genes in *M*. *smegmatis* that were sufficiently expressed. We noted that many (206) upstream boundaries of mapped RNA-seq reads aligned perfectly with a ribosome profiling boundary, and also coincided with a putative ATG or GTG (collectively, RTG) initiation codon (S1 Table). Of these RTGs, 130 (63.1%) matched the 5’ start of an annotated gene. This convergence of transcription and translation initiation is consistent with leaderless mRNAs (Fig 1A). The alignments of RNA-seq and ribosome footprinting boundaries that also align with an RTG codon in the *M*. *smegmatis* genome are collectively very stringent criteria. We observed 1412 RNA-seq boundaries, 2709 ribosome profiling boundaries, and 444,642 RTG occurrences in a genome of 13,976,418 bases (total for both strands), combining for a chance alignment genome-wide at 0.0087—far below the 206 we observed. Therefore, RTG codon occurrences are enriched at the 5’ boundaries of transcripts.

Our identification of abundant leaderless mRNAs in *M*. *smegmatis* lends empirical support for their presence in *M*. *tuberculosis* [9], as well as validating our experimental approach. A putative initiating RTG triplet at the other 76 RNA-seq and ribosome profiling 5’ boundaries identified unannotated start codons. Most corresponded to a new in-frame upstream (26) or downstream (28) start codon for an annotated gene, thereby extending or reducing the length of the annotated ORF, respectively (S1 Fig and S1 Table). The remaining 22 RTGs initiated new ORFs that had not been predicted by genome annotation pipelines (see below).

Leaderless transcripts generate distinctive 5’ read boundary profiles; their ribosomal footprints characteristically begin abruptly at the initiation codon. In addition, mRNAs with high ribosome occupancy can be enriched relative to their corresponding RNA-seq read depth in paired libraries that are otherwise comparable. Therefore, while some transcripts were not expressed highly enough to meet our read depth-mapping threshold in RNA-seq, ribosome isolation enriched those mRNAs enough to surpass the mapping threshold. Omitting the criterion of a coincident RNA-seq boundary from our analysis (still requiring a 5’ ribosome footprint aligning with an RTG) identified 762 candidate leaderless transcript boundaries. While we expect more false positives in this list of 762 transcripts, 457 (60.0%) matched a predicted annotated start, which was very similar to the 63% observed for the most stringent list, conferring confidence to those candidate initiation sites generated by this approach. Of the remaining 305 candidate leaderless RTGs, 175 (23%) were in-frame with annotated genes, predicting new upstream (99 instances) or downstream (76 instances) start codons for those ORFs (S2 Table). The 130 (17%) RTG codons that did not correspond to an annotated ORF represent potential initiation sites for new, unannotated, ORFs (S2 Table, column I). These metrics parallel our most stringent list above and suggest that 206 leaderless transcripts is an underestimate. Our *M*. *smegmatis* and *M*. *tuberculosis* transcript mapping data in its entirety can be interactively viewed by links found at <http://www.wadsworth.org/research/scientific-resources/interactive-genomics/>, and run directly through common internet browsers.

Leadered transcripts, however, generate a range of ribosomal footprints that begin in the 5’ UTR, spanning a putative Shine-Dalgarno site and the initiation codon (Fig 1B). The range of footprint created by the ribosome on the 5’ UTR creates ambiguity in confidently assigning the start codon. We reasoned that leaderless and leadered mRNAs would fundamentally differ in their RTG codon placement relative to the onset of the ribosome profiling footprint: leaderless RTGs would map to the 5’ ribosome profiling boundary, whereas ribosome occupancy would protect the 5’ UTR of leadered transcripts most proximal to the initiating RTG codon. We therefore plotted the distance between the 5’ ribosome profiling boundary and the next downstream RTG triplet as a candidate initiation codon. This generated two peaks: the major leaderless peak with no separation (762 candidates, as described above), and a second broad peak with a mode (103) at 24 nucleotides of separation (S2 Fig and S2 Table).

Conservatively bracketing the second broad peak to include spacing of 20 to 30 nt of separation between the footprint boundary and the next RTG, identified 731 candidate leadered initiation RTG codons. Of these, 423 (58%) mapped exactly to the start of an annotated gene, validating the predictive value of the leadered transcript dataset. Other RTG codons within this 20 to 30 nt footprint window were in-frame with the annotated ORFs, but were either upstream (45) or downstream (133) of the predicted start codon, indicating probable mis-assigned start codons. The remaining 130 RTG triplets within this ribosome footprint range could represent either initiation codons for new unannotated ORFs (as for the leaderless RTGs), or background internal RNA fragments that fortuitously include an RTG sequence.

### Independent demonstration of leaderless mRNAs in *M*. *tuberculosis* by TSS mapping and N-terminal mass spectrometry

We also took an independent approach in the related pathogenic species, *M*. *tuberculosis*, to determine whether non-canonical translation initiation mechanisms are conserved throughout the genus. We used a modified RNA-seq approach to map TSSs genome-wide, identifying 4,978 TSSs representing 2254 genes (S3 Table). We found that 1,098 TSSs (22%) initiated with an RTG triplet at the +1 position, and 497 (45%; S3 Table) of these corresponded to the annotated start codon of a gene, consistent with Cortes *et*. *al*. and our findings in *M*. *smegmatis*, that leaderless mRNAs are common in mycobacteria. Also consistent with our findings in *M*. *smegmatis*, an additional 76 (7%) and 208 (19%) extended or shortened the annotated reading frames of same-frame leaderless RTGs that were upstream or downstream of the predicted annotated initiation codon, respectively. These novel leaderless TSSs would be missed in studies that rely on the accuracy of annotated genomes.

Sites of translation initiation have been assumed from genome annotation predictions in *M*. *tuberculosis* [9], or determined by our ribosome-associated mRNA profiling (ribosome profiling) in *M*. *smegmatis*. We independently mapped sites of translation initiation in *M*. *tuberculosis* by a direct mass spectrometry (MS) approach focused on identifying the spectral signatures of those peptides with N-termini created by translation initiation, rather than tryptic digestion. We performed LC-MS/MS on tryptic digests of total protein from *M*. *tuberculosis* lysates and specifically searched for N-terminal peptides within annotated ORFs. ORFs that could be extended by conceptual translation to candidate initiation codons upstream were included in the search. To be confident of our N-terminal assignments, we then stringently limited our list to a very high-confidence subset of N-terminal peptides: those with acetylated N-terminal residues and those with intact methionines present at non-ATG codons. 211 protein N-termini met at least one of these criteria, 144 of which matched annotated starts (S4 Table). For leaderless genes, we predict that mapped N-termini should correspond to the +1 nucleotide of the TSS. Comparison of mapped N-termini and TSS addresses in *M*. *tuberculosis* confirmed translation initiation for 51 peptides (24%) that coincided with an RTG at the +1 position of the transcript (S4 Table). Thirty-two (62%) of these 51 leaderless *M*. *tuberculosis* N-terminus/mRNA pairs matched JCVI predicted genome annotations; a similar proportion to that predicted in *M*. *smegmatis* by ribosome-profiling (63%). Even though the reduced sensitivity (compared to our transcriptomic methods) of our N-terminal proteomic approach prevented a fully comprehensive survey of translation initiation sites, it provided independent, empirical evidence that leaderless mRNAs in *M*. *tuberculosis* are translated from the +1 nucleotide.

### Genomic features of leaderless and leadered mRNAs in mycobacteria

**Fig 2:**
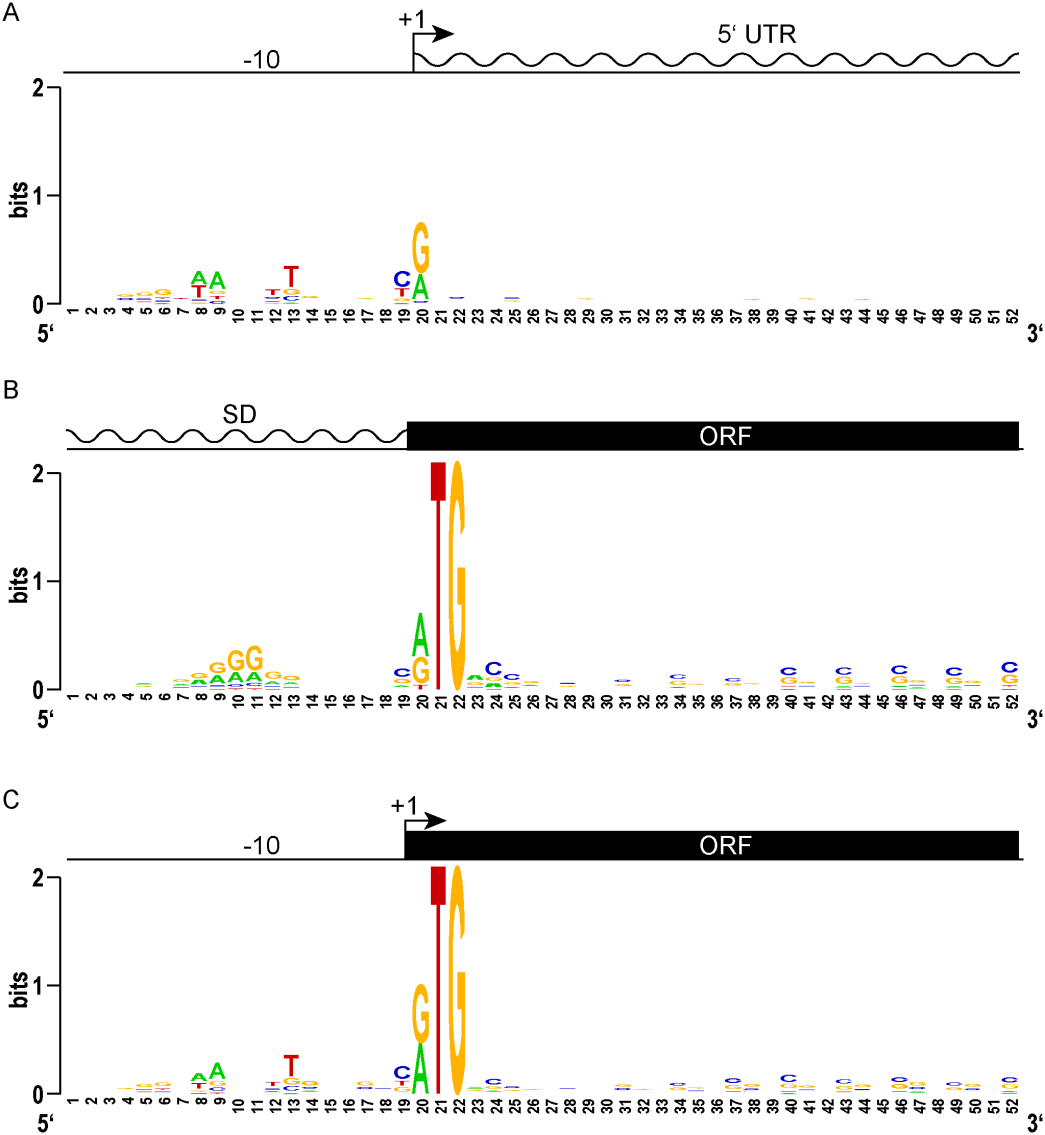
Leaderless gene architectures bring promoters and ORFs together. (A) Logo plot of TSS and proximal promoter region of traditional leadered genes. A purine (A or G) is favored at the +1 nucleotide, and an AT rich -10 element appears upstream. The 5’ UTR downstream of the transcription start site shows no sequence constraints or enrichment. (B) A Logo plot of the 5’ UTR from the translation initiation codon shows a Shine-Dalgarno-like AGGAGG sequence enrichment, centered 9–10 nt upstream (positions 10–11). From the initiation codon, the coding sequence downstream shows the wobble bias of the G-C rich mycobacterial genome. (C) The proximal promoter regions of leaderless genes have a -10 sequence of similar composition and spacing to that of leadered genes (compare to 2A). The TSS is also the first nucleotide of the translation initiation codon. There is no evidence of Shine-Dalgarno sequence enrichment upstream. The ORF initiated by leaderless codons shows the same wobble bias as seen in leadered ORFs.

We sought to identify genomic features that could distinguish the start sites for predicted leadered and leaderless genes. TSSs that did not have a candidate RTG initiation codon at the 5’ end were considered to represent classical leadered genes. An alignment of the 20 nucleotides upstream of the +1 transcription start site for the putative leadered transcripts (TSSs that began with something other than RTG) in *M*. *tuberculosis* showed the expected discernable enrichment for A and T nucleotides that mark the -10 hexamer for sigma factor binding (Fig 2A). Looking from these TSSs downstream into the putative 5’ UTRs showed no position-dependent enrichment of nucleotide sequences. Ribosome profiling in *M*. *smegmatis* allowed features around translation initiation codons to be examined. Viewing the 5’ UTR upstream from the translation initiation codon of the 731 transcripts with an RTG 20–30 nt from the 5’ ribosome profiling boundary (S2 Table) showed that these potentially leadered mRNAs were enriched for purines in the proximal upstream region where a Shine-Dalgarno element would be expected (Fig 2B). Slight displacement within the phasing tolerances separating the Shine-Dalgarno and initiation codon produced the mound of enriched purines, similar to the profile observed in *E*. *coli* [47]. Subsequent MEME analysis identified a Shine-Dalgarno-like core element in 318 of the transcripts (S3 Fig). The ORF initiated by the identified start codon was evident in downstream alignments by an enrichment of periodic C and G nucleotides, reflecting a selective pressure for the sequence of the encoded protein while conforming to the wobble codon bias of a G/C-rich organism. Therefore, the classic leadered transcripts in mycobacteria appear to have all of the hallmarks of bacterial genes: a -10 hexamer in the promoter, a Shine-Dalgarno sequence in the 5’ UTR proximal to the initiating RTG, and codon bias of the encoded open reading frame downstream of the RTG.

We reasoned that the features of leaderless genes should be very similar to the leadered mRNAs, though lacking the features found in 5’ UTRs. Alignment and Logo projection of the sequences upstream of *M*. *tuberculosis* RTG-initiated TSSs showed a -10 hexamer that was indistinguishable from that of leadered genes (Fig 2C). This observation underscores the presence of a promoter upstream of the mRNA’s +1 site, and the lack of a Shine-Dalgarno element upstream of the RTG translation initiation codon. These genomic features provide independent evidence that leaderless transcripts are generated *de novo* by transcription, and not by post-transcriptional processing. As observed for the classic leadered ORFs, the synchronous wobble of the leaderless ORFs suggests that the encoded proteins have experienced similar selective pressures as leadered genes.

Our comparative genomic analyses between leaderless and leadered genes did not identify any new motifs specifically associated with leaderless gene expression. However, the convergence of canonical promoter elements juxtaposed with protein coding sequences without intervening untranslated regions again supports our conclusion that there is widespread leaderless translation.

### Conservation of non-canonical translation initiation between *M*. *smegmatis* and *M*. *tuberculosis*

We compared orthologous *M*. *smegmatis* and *M*. *tuberculosis* genes to assess the conservation of leaderless gene structures. Of the 206 *M*. *smegmatis* leaderless transcripts, 184 corresponded to annotated genes, with the leaderless RTG representing the predicted start (130 instances) or a new start either upstream or downstream of the predicted start (54 instances). Of these, 114 of had orthologs in *M*. *tuberculosis* (S1 Table), most (97) of which had TSSs within 200 nt of the annotated start, suggesting that they were sufficiently expressed for analysis. Of these 97 expressed *M*. *tuberculosis* genes, 71 were also leaderless in this species. Therefore, ~73% (71/97) of *M*. *smegmatis* leaderless genes have expressed leaderless orthologs in *M*. *tuberculosis*. Orthologs with conserved leaderless initiation codons highlight the need for empirical reannotation of these genomes (see example, S4 Fig and see below).

### Targeted reporter assays for leaderless translation initiation

**Fig 3:**
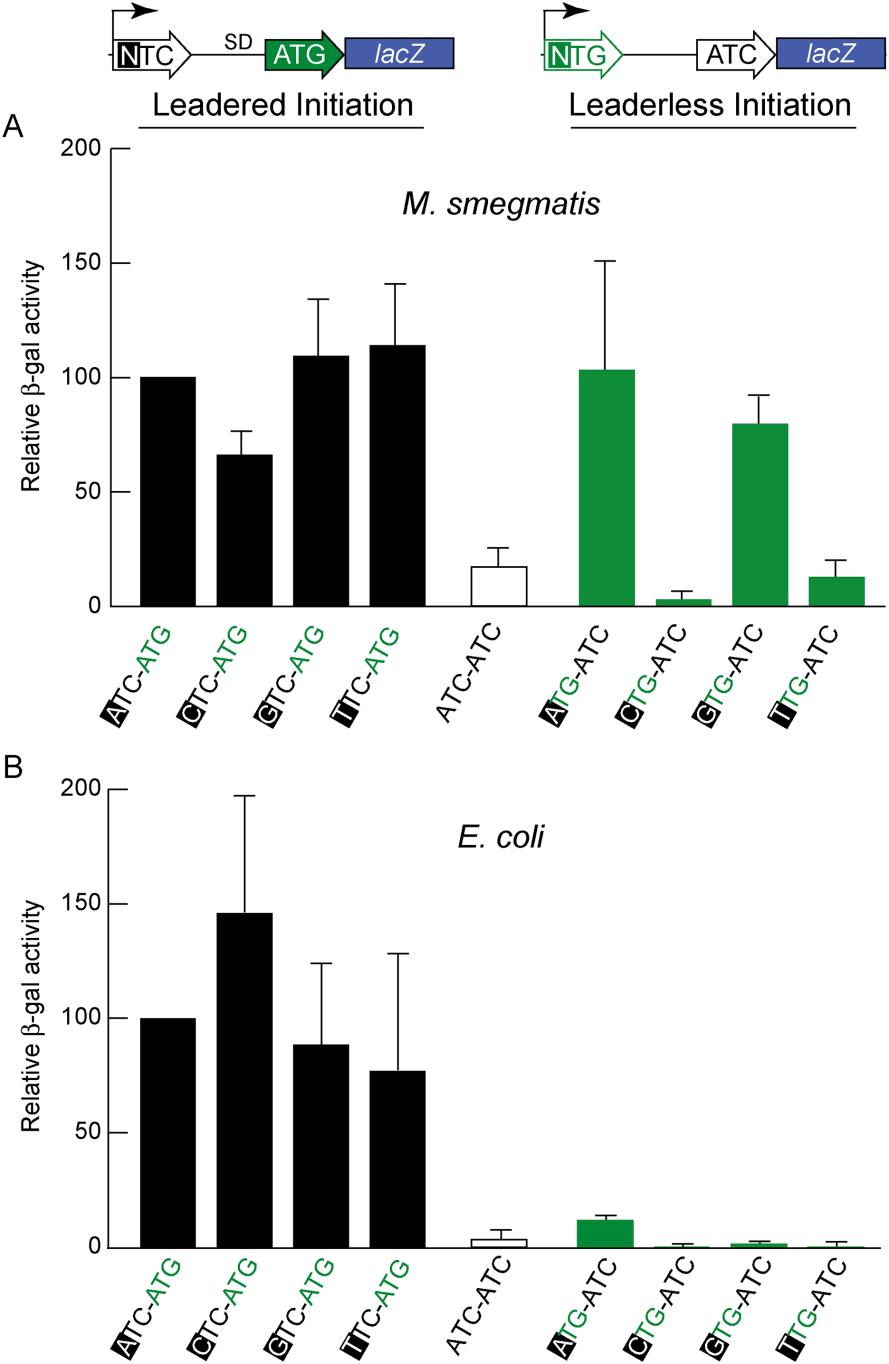
Directed β-galactosidase reporters show that leaderless ATG and GTG triplets initiate robust translation of *lacZ* in *M*. *smegmatis* (A) but not in *E*. *coli* (B). Candidate codons were tested in leadered and leaderless contexts for their activity in initiating translation of the adjacent *lacZ* gene. Putative positive (ATG) and negative (ATC) controls provided reference activities. The level of β-galactosidase expression in *M*. *smegmatis* from leaderless transcripts (upper right green bars beginning with an ATG or GTG codon) was similar to that from leadered transcripts (upper left black bars). CTG and TTG candidate codons were ineffective initiators at the leaderless position. Differences in β -galactosidase activities in *M*. *smegmatis* leaderless constructs were not due to effects of the +1 nucleotide (indicated by black background) on transcript abundance, as corresponding leadered constructs were not comparably affected.

Genomic analyses provide a broad overview of translation initiation sites, but encompass too many variables that obscure mechanistic insight. To control for some of the variables associated with leaderless translation, we developed reporter constructs that allowed β-galactosidase quantification of translation efficiency directed by candidate leaderless and leadered mRNAs (Fig 3). N-terminal fusions have negligible effects on β-galactosidase activity [48]; therefore, translation of the 5’ leader should be well tolerated. In a fixed reporter context, translational features were independently tested, including the presence of an initiation-competent start codon (e.g., ATG), or a putative initiation-incompetent codon (e.g., ATC) at either the leadered or leaderless positions.

In *E*. *coli*, β-galactosidase activity supported through leaderless translation initiation was ~10% of that observed through leadered ATG initiation (Fig 3B); none of the candidate NTG initiation codons were able to support substantial translation from the leaderless position. When introduced into *M*. *smegmatis*, these same leaderless reporters were translated as efficiently as leadered constructs (Fig 3A); transcripts with an ATG or GTG beginning at +1 produced β-galactosidase activity levels similar to an ATG at the leadered position. The CTG and TTG candidate codons were poor initiators at the leaderless position, consistent with our genomic data that showed no enrichment of these triplets in *M*. *tuberculosis* transcription start sites, or ribosome occupancy by ribosome profiling in *M*. *smegmatis*. Our TSS mapping for *M*. *tuberculosis* indicated that transcription initiation favors purines at the +1 position (95% of TSSs), and the +1 nucleotide identity could influence promoter strength as it does for T7 RNA polymerase [49]. Thus, there was a possibility that β-galactosidase activity differences could arise from the effect of different +1 nucleotides on the robustness of transcription, rather than translation. To ensure that changing the +1 nucleotide did not affect transcript abundance, each nucleotide was tested at the +1 position for its effect on β-galactosidase activity produced by a conventional leadered ATG codon. The levels of β-galactosidase activity indicated only a slight effect of a +1 pyrimidine on mRNA levels (Fig 3A). We conclude that ATG or GTG, but not CTG or TTG, codons directed leaderless translation in mycobacteria at levels comparable to a leadered ATG codon in this context.

### Unbiased selection defines the sequence requirements for leaderless translation in mycobacteria

**Fig 4:**
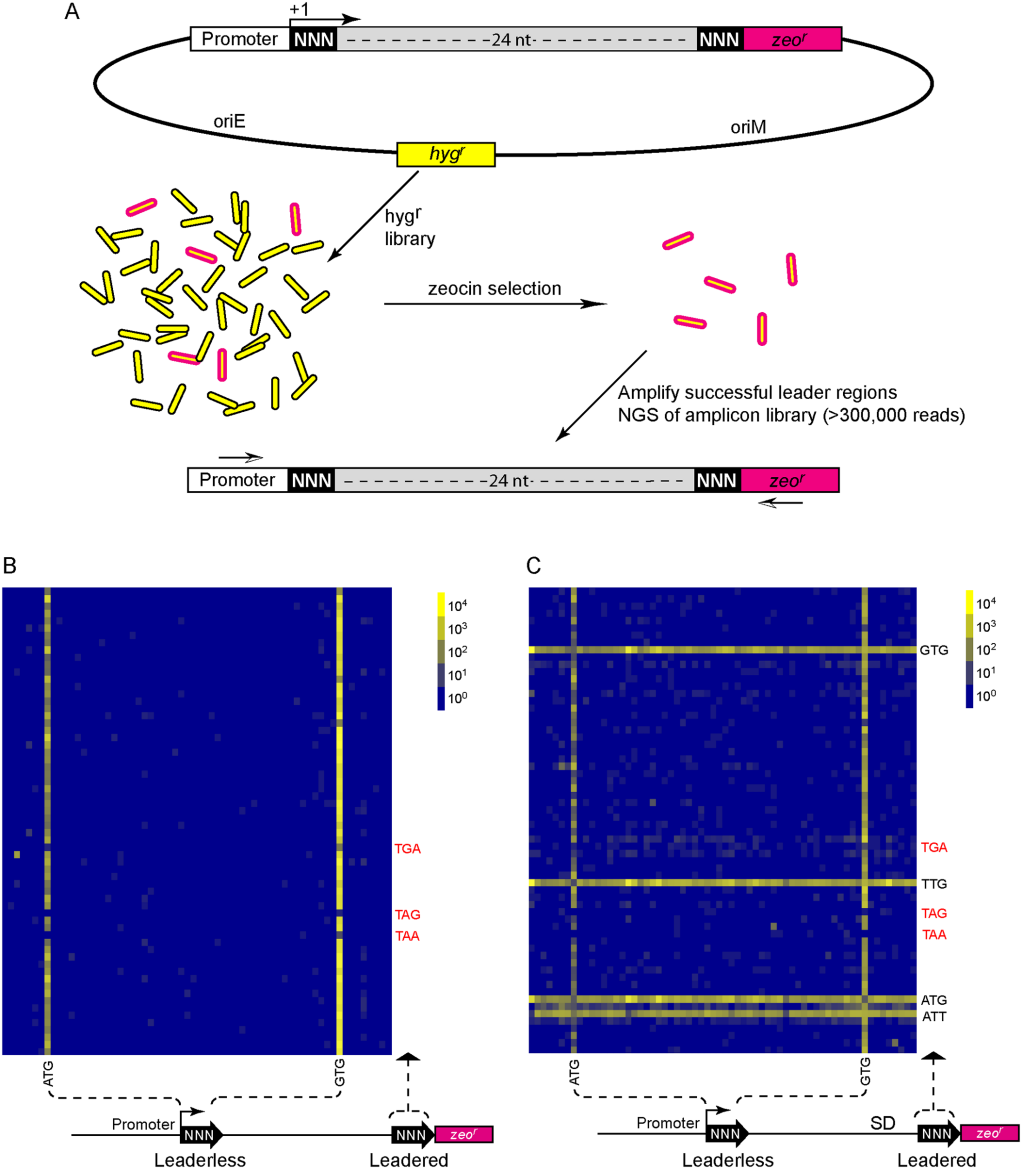
A translational reporter system identified leaderless and leadered initiation codon preferences. (A) Libraries of leader sequences were generated using two overlapping oligonucleotides, each with a single randomized codon positioned either at the leaderless position (+1) or the leadered position (+30), in-frame with the zeocin-resistance (*zeo* ^r^) gene. Self-primed heterodimers were inserted between the promoter and the *zeo* ^r^ gene and transformed into *E*. *coli*. The library was electroporated into *M*. *smegmatis*. Hygromycin selection allowed maintenance of the complete library, while zeocin selection required translation initiation at either one of the randomized codon sites. Following selection in zeocin, plasmids were recovered and the leader regions amplified for Ion-Torrent sequencing. Deep sequencing of amplicon libraries allowed the unbiased identification and estimation of relative efficiency of initiation codons. (B) A Shine-Dalgarno site was omitted to facilitate direct comparison between leaderless and leadered architectures. Read counts were compiled for each of the 64 possible codons at the leaderless position (columns) and leadered position (rows). Heat map indicates read counts of each combinatorial leaderless/leadered codon pair, from 10^0^ (blue) through 10^4^ (yellow). Only ATG or GTG at the leaderless position were capable of initiating translation of *zeo* ^r^. At the leadered codon position, no enrichment indicated that translation initiation did not occur at any of the possible codons. A further reduction of the expected stop codons suggested that they prevented read through of leaderless ribosomes into the *zeo* ^*r*^ ORF. (C) A Shine-Dalgarno sequence enables efficient use of diverse leadered initiation codons. A consensus Shine-Dalgarno (SD) element was placed upstream of the randomized leadered codon position. Zeocin-resistant pools showed a complex pattern of active translation initiation codons at both the leaderless and leadered positions. The presence of a Shine-Dalgarno supported translation initiation activity of ATG and GTG triplets in the leadered position, as well as the less common TTG and ATT triplets.

We then took an unbiased approach to identify sequences that can initiate, or influence the initiation of, translation. We developed a viability reporter predicated on sufficient translation of a zeocin antibiotic resistance gene (*zeo* ^*r*^) to allow selection of sequences that could successfully initiate translation, which could then be identified by PCR amplification followed by next generation sequencing (Fig 4A). Clusters of randomized nucleotides were embedded in the 5’ leader to address specific hypotheses. The pre-selection total library was maintained by hygromycin selection, encoded elsewhere in the plasmid. Growth in culture with zeocin antibiotic required translation of the zeocin reporter, directed by sequences present in the 5’ leader.

A library was generated with randomized nucleotides comprising the leaderless codon (5’ end of transcript) and at the annotated leadered start codon of the *zeo* ^r^ ORF in order to create constructs that could potentially support translation initiation from either of these two codons. The Shine-Dalgarno sequence was omitted. Sequencing amplicon pools of the zeocin resistant *M*. *smegmatis* showed that an ATG or GTG beginning at the +1 position of the transcript was necessary to initiate translation of the downstream *zeo* ^r^ gene (Fig 4B). Leaderless RTG reads accounted for all but 547 of the 343,242 total reads (S5 Table). Importantly, initiation by RTG codons at the leaderless position was independent of codon identity at the leadered position, with the notable exceptions of the three possible stop codons. No codon at the leadered initiation position was consistently enriched, indicating an absolute requirement for an appropriately placed Shine-Dalgarno sequence for efficient leadered translation initiation in our reporter constructs.

We then repeated the assay in the context of a consensus Shine-Dalgarno sequence. The zeocin resistant pool now showed strong enrichment for specific sequences at both the leaderless and leadered start codons (Fig 4C). ATG and GTG were again enriched at the leaderless start codon position, accounting for 17.0% of the 299,098 total reads (S5 Table). In contrast to reporters lacking a Shine-Dalgarno, ATG (20.5%), GTG (22.2%) and TTG (28.7%) were now enriched at the leadered start codon position (net leadered total 245,065), even more robustly than at the RTG leaderless initiation codons. Notably, CTG (0.03%) was not enriched, consistent with the paucity of CTG start codons in mycobacteria. Surprisingly, ATT (9.8%) codons were substantially enriched at the leadered position, indicating that this codon can act as a translation initiation codon in this context in mycobacteria. We note that our presumed negative control initiation codon, ATC, persisted through zeocin selection (0.2%), indicating low, but above background, translation initiation activity when accompanied by a Shine-Dalgarno sequence.

**Fig 5:**
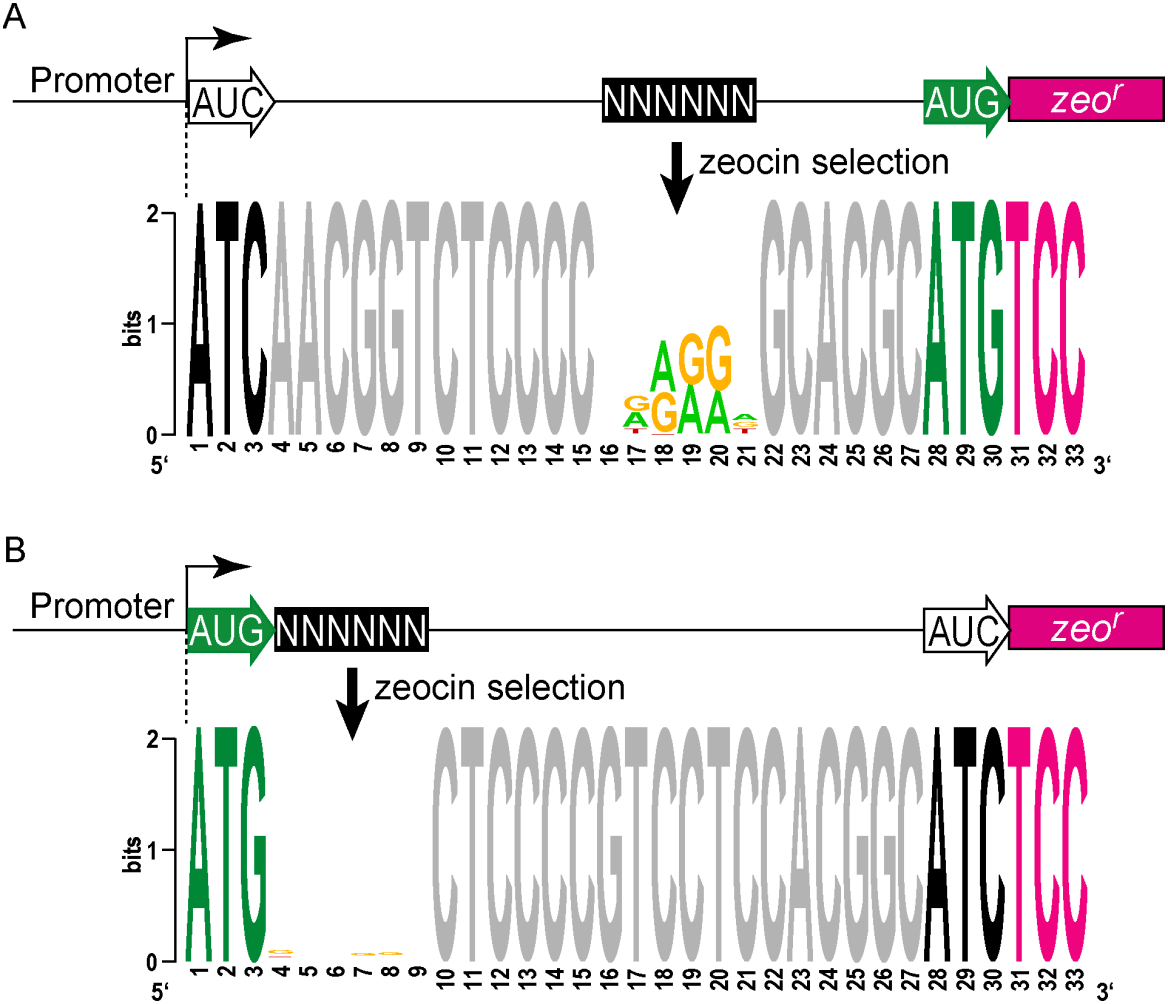
Definition of *cis* elements that support translation initiation in mycobacteria. (A) Zeo-seq viability reporter libraries were generated to determine the sequence context preferences for a SD upstream of a leadered initiation codon. Randomized nucleotides were positioned upstream of a leadered initiation codon, and zeocin selection enriched for Shine-Dalgarno-like sequences, indicating that mycobacteria adhere to this canonical translation criterion. (B) Leaderless translation initiation exhibits no sequence preference in the adjacent mRNA. A block of 6 nt was randomized immediately downstream of a leaderless initiation zeocin reporter construct. Sequences in the recovered pools of zeocin-resistant *M*. *smegmatis* were not enriched in composition or motifs in this region. The absence of any detectable enrichment in the randomized region for the leaderless pool indicates that there are no nucleotide preferences for efficient leaderless initiation in mycobacteria downstream of the RTG codon.

We modified the unbiased zeocin resistance selection approach to define sequences suitable for ribosome binding in mycobacteria. A contiguous block of 6 nt was randomized upstream of a leadered ATG initiation codon, and constructs providing zeocin resistance were collected and sequenced (S5 Table). We found sequences enriched for adenines and guanines in the randomized region, typical for a canonical Shine-Dalgarno site (Fig 5A). This is consistent with the conservation in mycobacteria of the complementary anti-Shine-Dalgarno element on the 16S rRNA.

We further leveraged our viability reporter system to determine whether any additional sequences support translation of leaderless transcripts near the ATG or GTG codon at the +1 position. Six contiguous random nucleotides were embedded immediately downstream (positions +4 through +9) of a leaderless ATG triplet (S5 Table). All transcripts had an ATC triplet in the leadered position, and lacked the necessary Shine-Dalgarno sequence, thereby precluding leadered initiation and requiring leaderless translation initiation for zeocin resistance. The selected library pools showed no discernable enrichment of motifs or nucleotide composition (Fig 5B). This finding was consistent with the lack of enrichment for any specific downstream sequence associated with naturally occurring leaderless transcripts (Fig 2C).

### Mycobacteria may encode hundreds of small proteins

Our ribosome profiling in *M*. *smegmatis*, and our viability reporter assays, indicated that an RTG triplet at the TSS is necessary and sufficient to direct leaderless translation initiation. Therefore, leaderless +1 RTGs not initiating translation of annotated reading frames are initiating translation of novel ORFs (S1 Fig). Our most conservative leaderless dataset from *M*. *smegmatis* requiring the convergence of three parameters (RNA-seq boundary, ribosome profiling boundary, and an RTG triplet), identified 22 unannotated ORFs (S1 Table). We also identified 130 RTG codons (of 762) at the 5’ boundary of the ribosome profile consistent with their initiation of unannotated ORFs (see the leaderless peak in S2 Fig and S2 Table, column H). Ribosome profiling of these novel ORFs showed association with 70S ribosomes, indicating that they are very likely to be translated. These novel ORFs often encode small proteins (defined here as 5–50 amino acids) that map immediately upstream of an annotated ORF (S6 Table). While the size limits applied to this definition have not been functionally determined, the range highlights a collection of proteins often excluded in annotation prediction algorithms. These encoded small proteins could function as diffusible products in *trans*, as do larger sized proteins; alternatively, they could act in *cis*, influencing the expression of downstream ORF(s). Our data suggest two *cis* mechanisms by which the ORF, rather than the encoded small protein, may regulate the downstream genes of the polycistronic mRNA (see below).

**Fig 6:**
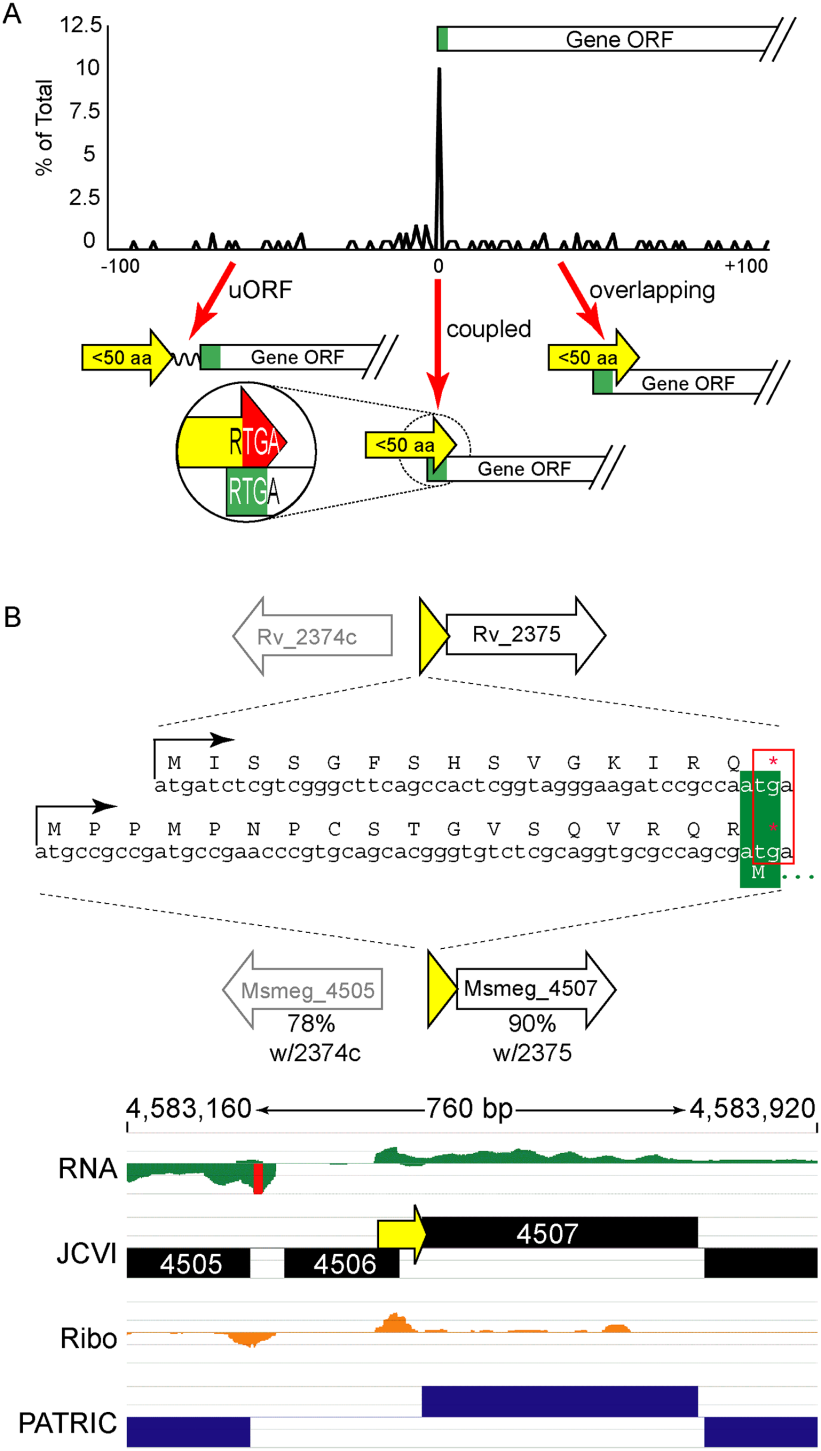
Small protein ORFs are frequently coupled to the ORF downstream. (A) *M*. *tuberculosis* leaderless transcripts initiate unannotated small protein ORFs that terminate at the start of the annotated gene downstream more often than expected. All small protein ORF stop codons within 100 nucleotides of an annotated gene start are shown relative to that start codon (0 = coupled RTGA overlap). Three structural classes are identified: uORFs (the small ORF terminates upstream of the annotated start), coupled ORFs (linked by an RTGA tetramer), and overlapping ORFs. The y-axis shows the fraction of small ORFs that terminate a specified distance (x-axis) from the annotated start codon of the downstream gene. (B) One example of a coupled small protein in *M*. *tuberculosis* and *M*. *smegmatis*, upstream of orthologous genes. The primary sequence of the encoded small protein is not conserved, but the leaderless initiation and coupled linkage is maintained.

The ORFs encoding small proteins can be categorized with respect to the position of their stop codon relative to the initiation codon of the adjacent annotated gene (Fig 6A and S1B Fig). The first class comprises proteins whose ORFs stopped upstream of the neighboring annotated gene. We refer to these as upstream ORFs (uORFs), a nomenclature applied extensively to equivalent ORFs in eukaryotes. The second class comprises proteins whose ORFs extend into the coding region of the annotated ORF downstream, utilizing an alternative reading frame (overlapping ORFs). A subset of this class comprises proteins for which the TGA stop codon of the upstream peptide also formed the second two nucleotides of the RTG start codon of the downstream ORF; we refer to these small proteins as “coupled”.

We find similar numbers of predicted small proteins are encoded from leaderless transcripts in *M*. *tuberculosis* using an entirely different experimental approach. Our TSS data indicated that 317 (28.8%) of the leaderless RTG codons initiated novel ORFs, and 197 of these are between 5–50 amino acids suggesting that *M*. *tuberculosis* encodes hundreds of undocumented small proteins (S3 Table, see “Leaderless” sheet, column K). Forty-nine of these small ORFs appear to be encoded on transcripts separate from annotated genes under the conditions analyzed, while others appear to be encoded polycistronically and followed by annotated genes. Collectively, our data are consistent with mycobacterial small proteomes comprising hundreds of unannotated small proteins.

Coupled small proteins require a precise overlap of two codons (one Opal stop, one start). Our data indicate that these structures are significantly overrepresented in mycobacteria (Fig 6A, Fisher’s Exact Test p < 0.0003). The RTGA sequence links the two ORFs physically, and likely has functional consequences resulting from coupled translation. Sometimes only the genomic placement and coupled architecture of divergent small proteins are conserved between *M*. *tuberculosis* and *M*. *smegmatis*, suggesting that the coupled linkage is under greater selection than the encoded small protein sequence (see example Fig 6B). The relative contribution of coupling to the total translation initiation of the downstream ORF may augment overall gene expression by maximizing ribosome occupancy.

**Fig 7:**
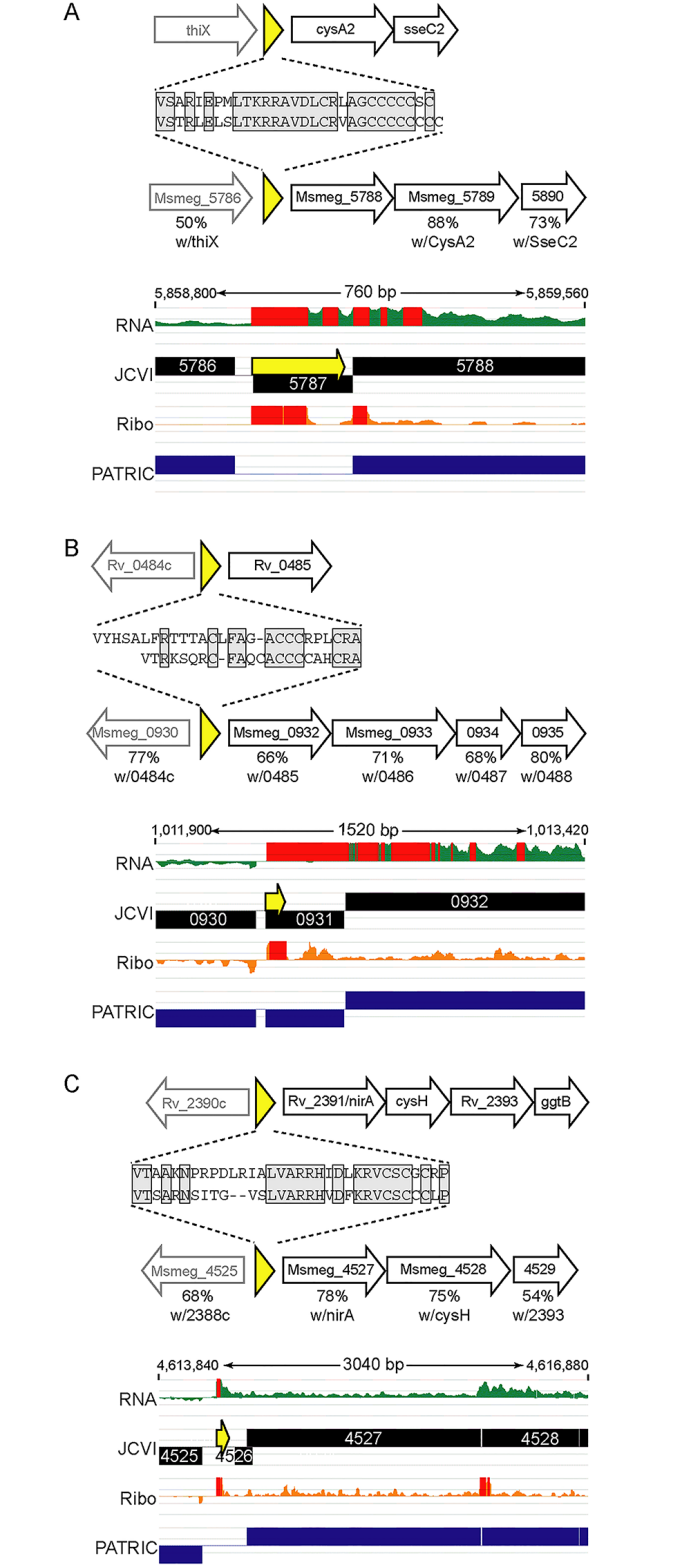
Examples of conserved small proteins encoded by leaderless mRNAs in mycobacteria. Small ORFs were identified upstream of annotated orthologous genes (A) *cysA2*/Msmeg_5788, (B) Rv0485/Msmeg_0932, and (C) *nirA*/Msmeg_4527, in the *M*. *tuberculosis* and *M*. *smegmatis* genomes. Schematic representation of loci in *M*. *tuberculosis* (above) or *M*. *smegmatis* (below) that encode small proteins (yellow) upstream of genes conserved between these species. The deduced amino acid sequence of each small protein is shown, with the conserved amino acids in gray shaded boxes. The genes downstream in black block arrows are putative members of the same mRNA, and the gene designated by the gray arrow upstream is transcriptionally independent, but shown for context. The amino acid identity with the protein encoded by the corresponding *M*. *tuberculosis* gene is indicated below the respective *M*. *smegmatis* gene. Below are screen shots of RNA-seq and ribosome profiling profiles in *M*. *smegmatis*, and annotated gene predictions from two different annotation algorithms JCVI (black) and PATRIC (blue). Small proteins encoded throughout genomes are poorly annotated; note here that pipeline annotation algorithms predicted none of the small proteins, and in some cases predicted longer proteins on the opposite strand for which we see no transcriptional or translational evidence.

We identified orthologous uORFs in *M*. *smegmatis* and *M*. *tuberculosis* (Fig 7). These small proteins shared not only their genomic context, but also significant amino acid identity. The juxtaposition of the encoded small proteins and downstream genes suggests an operonic structure, with possible regulatory effects. Small uORFs in other bacterial species are often found at the 5’ end of polycistronic mRNAs dedicated to biosynthetic pathways for amino acids, wherein the amino acid product of the biosynthetic operon is overrepresented in the encoded peptide. An abundance of that amino acid transcriptionally attenuates the downstream biosynthetic genes in the operon. Inspection of the encoded peptides for enrichment of amino acids identified several candidates for attenuating peptides. One predicted small ORF contained a cluster of codons for cysteine at its C-terminus, and was located upstream of the *cysA2* gene, Rv0815 (Fig 7A). The relative location and sequence of this encoded protein is conserved in mycobacteria, strongly supporting its identification as a functional small ORF. Our ribosome profiling of the orthologous locus in *M*. *smegmatis* showed robust 70S ribosome occupancy of both the small ORF and the downstream genes (Fig 7A). We speculate that cysteine homeostasis or redox status may influence ribosome processivity through the small ORF, affecting the transcription or translation of the linked operon genes downstream. We found other upstream-encoded small proteins that were conserved in mycobacteria, and enriched in cysteine residues upstream of putative hypothetical or cysteine-metabolic genes (Fig 7B and 7C, respectively). We note that in each of the examples shown, the JCVI annotation predicted a gene encoded on the opposite (negative) strand to these uORFs; our data find no negative-strand transcripts to support those predicted annotations. These examples illustrate the functional potential of a few of the members of the mycobacterial small proteome, providing testable hypotheses for these candidates (e.g., through cysteine or redox modulation), while others may have less obvious—but no less important functional roles. The location of many unannotated uORFs as the first genes in their operons, suggests new possibilities for regulatory and functional interactions of the small proteins with the downstream operon and encoded proteins.

## Discussion

We combined independent assays from two species to profile the transcriptional and translational landscapes of mycobacteria. Our findings collectively indicate that translational mechanisms and the gene architectures they process differ from the canonical models derived from extensive studies in *E*. *coli*. A prominent non-canonical feature is the frequency, translational robustness, and conservation of leaderless mRNAs in mycobacteria. Experimentally determining translation initiation sites of actively translated ORFs provides empirical evidence for the prevalence of mycobacterial leaderless transcripts, and significantly refines and extends leaderless gene predictions derived from genome annotations [9]. We find 2166 TSSs also identified by Cortes et al. in addition to many novel initiation sites, while our independent *M*. *smegmatis* approach provides evolutionary depth in a tractable model bacterium. There does not appear to be a unifying criterion for leadered and leaderless gene structures. They have comparable GC content, initiate genes of similar length, begin operons of similar composition, and—under standard growth conditions—are not enriched for functionally related proteins.

We developed novel reporters to begin to address mechanistic hypotheses about *cis*-sequences that affect leaderless and leadered translation initiation in mycobacteria. Our data show that any mRNA beginning with ATG or GTG (AUG or GUG in mRNA) will be translated as a leaderless mRNA in mycobacteria. Strikingly, no other codon initiates leaderless translation, in contrast to leadered translation, which can initiate with a wide variety of codons, including rare start codons such as TTG and ATT. In addition to Shine-Dalgarno dependence, differing start codon requirements indicate fundamental mechanistic differences between leadered and leaderless translation initiation. However, there is currently insufficient understanding of leaderless translation to determine why this difference exists.

The combination of multiple, independent observations strongly indicate that a +1 RTG codon is both necessary and sufficient for leaderless translation initiation. We did not identify any sequences beyond the start codon that were enriched in leaderless genes, either in mycobacterial genomes or by our viability reporter assays, suggesting that the RTG start codon is not supported by an enhancing downstream box [7]. Leaderless genes in *M*. *smegmatis* are frequently leaderless in *M*. *tuberculosis*, in spite of sequence variation (indicating an absence of a functionally important element) near the initiating RTG codon. We observe robust leaderless initiation regardless of the reporter context, none of which have mycobacterial origins that could contain leaderless support elements. Varied contexts and disparate assays consistently produce robust leaderless translation efficiencies in mycobacteria. Thus, an ATG or GTG at the TSS is the only discernable criterion for translation initiation at that same site.

Previous studies in *E*. *coli* have indicated that ATG was optimal for leaderless initiation, with <10% activity for GTG [27]. Moreover, leaderless expression from even an optimal ATG initiation codon in *E*. *coli* is weak (10%) compared to initiation from a leadered transcript [41 and Fig 3], again contrasting to the relatively similar levels of expression from leadered and leaderless mRNAs in *M*. *smegmatis*. Together, these observations indicate that recognition and translational initiation of leaderless mRNAs in *M*. *smegmatis* occurs by a distinctly different mechanism than that described for *E*. *coli*. Overall leaderless gene expression appears to be controlled by a balance of promoter activity, mRNA stability, and protein turnover; leadered gene expression has an additional level of control provided by features of the 5’ UTR, including the Shine-Dalgarno sequence and RNA secondary structure. We speculate that this additional level of expression modulation may have provided the evolutionary impetus for leadered gene structures.

Analyses of TSS mapping data indicate that RNA polymerase exhibits context bias in selecting a TSS. For example, a cytosine at the -1 is preferred, followed by a pyrimidine at the +2 [50]. Could RNA polymerase sequence preferences be responsible for driving the enrichment of RTG (1098/4979, 22%) triplets at the transcription start sites of *M*. *tuberculosis*? Substituting chemically similar but translationally inert nucleotides into the second and third positions of the RTG triplet, we see that they are found at TSSs much less often: RCG is found at 270 (5.4%) of *M*. *tuberculosis* TSSs, and RTA at 121 (2.4%). Clearly, the over-representation of RTG at TSSs (Fisher’s Exact Test, p <0.0001) indicates that there is a strong evolutionary selection for RTGs at the TSS in mycobacteria.

We conservatively defined leaderless genes as those that have an RTG beginning at the first (+1) nucleotide of their corresponding transcript. Leaderless genes have also been more broadly defined as those having a potential initiation codon from +1 through +5 [9], with the rationale that a miniscule 5’ UTR could not include a Shine-Dalgarno element. Convergence mapping of our protein N-termini and *M*. *tuberculosis* transcriptional start sites provides some experimental assessment of the accuracy of this broader definition. While N-termini were identified for 51 of 1098 (4.7%) leaderless +1 TSSs, only 4 of 558 (0.7%) candidate RTG codons from +2 to +5 had associated N-termini (S4 Table and S5 Fig). Many factors affect peptide identification in mass spectrometry analyses; therefore the relative paucity of N-terminal peptides initiated by +2 to +5 leaderless codons is not definitive. Nevertheless, our data suggest that translation initiation efficiency is greater for RTG codons at +1 than from +2 through +5. Independent of the N-terminal mapping, the non-random distribution of RTG triplets in the first five positions of *M*. *tuberculosis* transcripts supports RTG enrichment specifically at the +1 nucleotide. If leaderless translation initiation utilizes any near-TSS RTG equivalently, RTGs should be evenly distributed throughout this window. However, the 1098 RTG triplets at the +1 are greater than the next four positions combined (558) (S5 Fig). Including RTG codons downstream of the +1 position in the definition of leaderless mRNAs should therefore carry the caveat that their initiation efficiencies are likely to be lower than an absolute leaderless codon. The enrichment of RTGs at the TSS in mycobacteria clearly indicates a positive selection for leaderless translation in the genus, and not simply a tolerated inefficiency of a promiscuous initiation mechanism.

Leaderless translation is far more efficient in *M*. *smegmatis*, and presumably *M*. *tuberculosis*, than in *E*. *coli*. The reason for this disparity is unclear, although there are many differences in ribosomal proteins between mycobacteria and *E*. *coli* [51]. Importantly, almost all studies of leaderless translation have used *E*. *coli* as a model system. As illustrated in our genomic landscape profiles, the mutually exclusive presence of a -10 hexamer or a Shine-Dalgarno ribosome-binding site upstream of the initiation codon can distinguish leaderless from leadered genes. Our data suggest that other species with a high frequency of predicted leaderless gene structures [21, 22] are also likely to initiate leaderless translation efficiently and by a different initiation mechanism than that for *E*. *coli*.

The unbiased sequence-panning approach we developed to define *cis* elements that support translation initiation clearly show that a Shine-Dalgarno sequence is required for efficient leadered initiation. The purine-rich consensus of the recovered zeocin-resistant constructs agreed well with the canonical motif defined in *E*. *coli* [1, 47]. The functionally selected ribosome binding sites also agreed well with our genomic sequence analyses of the 5’ UTRs of leadered transcripts in *M*. *smegmatis* (DNA Logo in Fig 2B and S3 Fig). The conservation of canonical Shine-Dalgarno elements in 5’ UTRs and cognate anti-Shine-Dalgarno sequences in 16S rRNA tails, suggests that the mechanism aligning the ribosomal subunits with a leadered translation initiation codon in these two species is conserved.

Our focus on structurally and functionally defining the 5’ ends of genes led us to identify large numbers of small ORFs that are likely to be translated in mycobacteria. The prevalence and efficiency of leaderless translation initiation that we see in mycobacteria support the prediction that leaderless ORFs will be translated. It is possible that upstream small ORFs enhance translation initiation of the annotated genes downstream, particularly in cases of RTGA coupling. This may be through non-canonical re-initiation of 70S ribosomes that remain intact while tracking into the downstream ORF. Alternatively, traditional Shine-Dalgarno mediated initiation of the downstream ORF may be augmented by an increased local concentration of ribosomal subunits at the junction of the two ORFs. Previous studies of re-initiation have focused exclusively on pairs of ORFs within operons. Operons comprise annotated ORFs that are frequently coupled at their junctions as well, which may offer efficiency advantages through ribosome loading of the downstream gene. Alignments of annotated operon gene junctions clearly show that wobble positions of the codons are guanine/adenine enriched for dual use as a Shine-Dalgarno sequence embedded in coding sequence (S6 Fig). This suggests that translation initiation of the downstream gene can occur by two distinct mechanisms (non-canonical re-initiation, and canonical Shine-Dalgarno directed), or that a Shine-Dalgarno sequence can improve the efficiency of re-initiation. Similar mechanisms may apply to the couplings joining small protein ORFs with their downstream genes as described here. The delivery of ribosomes to the downstream ORF via a coupling tetramer does not depend on the amino acid sequence of the small protein sequence itself, only that, once loaded, the ribosomes make it through that ORF to be near the initiation codon of the downstream ORF. It is possible, however, that the sequence of the encoded peptide might modulate ribosome delivery.

The conserved candidate cysteine-rich uORFs that we identified would be the first examples of attenuation identified in mycobacteria, and for cysteine-directed attenuation in bacteria. While our hypothesized *cis* regulatory roles for the small protein ORFs are still speculative, they may offer an alternative regulatory mechanism compensating for the loss of the 5’ UTR in leaderless genes. These possible mechanisms could help modulate protein production beyond promoter-controlled transcription initiation.

The precedent and the presence of potential *cis* regulatory ORFs are clearest for small proteins encoded at the beginning of an operon[43, 52–54]. Nevertheless, precedent also exists for *trans* activities inherent to the small proteins themselves[54]. It is tempting to speculate that upstream leaders are fertile incubators poised for the nascent evolution of expressed sequences as the first gene of an expanding operon. Moreover, some of the predicted small proteins are not apparently affiliated with another nearby ORF (at least 49 in *M*. *tuberculosis*), suggesting that these small protein singletons are not *cis*-regulators. It is unlikely that every member of the mycobacterial small proteome has an individual function, but clearly some of them bear hallmarks of functional features. Small protein ORFs that are part of an operon may function in the pathway encoded by the trailing genes of that operon. Expanded study of the emerging small proteome will help to identify the likely candidates, determine their functional roles, and evaluate their collective influence on the phenotype or phenotypic variation of the bacteria.

We focus on small proteins encoded by leaderless transcripts because of the abundant support for their presence in both mycobacterial species analyzed here. However, as leadered initiation of annotated genes in mycobacteria still outnumber leaderless annotated genes by at least two to one, it is reasonable to speculate that leadered codons will initiate translation of small proteins at comparable rates. Therefore, the small proteome estimates derived solely from leaderless mRNAs under standard rich media growth conditions, as presented here, are conservative underestimates.

In summary, we have identified widespread, conserved, non-canonical translation initiation in mycobacteria. Abundant leaderless translation initiation, encoded small proteins, and coupled linkages suggest translation mechanisms in mycobacteria are likely to fundamentally differ from those modeled in *E*. *coli*. Further, our data may help improve current genome annotation pipelines to more accurately predict leaderless gene structures, transcription and translation isoforms, and translation initiation sites in bacteria. Moreover, we anticipate that genome-scale approaches combining ribosome profiling, TSS, and LC-MS/MS will uncover similar phenomena in diverse bacterial species, challenging long-held paradigms of translation initiation in bacteria.

## Materials and Methods

### Mycobacteria and growth conditions

The mc^2^155 strain of *M*. *smegmatis* was grown in Middlebrook 7H9 broth supplemented with ADC and 0.05% Tween 80 with shaking at 230 RPM at 37°C to an OD_600_ of ~1.0. *Mycobacterium tuberculosis* (strain H37Rv) was cultured in Middlebrook 7H9 supplemented with OADC, 0.05% Tween 80 and 0.2% glycerol to an OD_600_ of 0.8–1.0 for transcriptomics experiments, and the same strain was cultured as described in [55] for mass spectrometry.

### Extract preparation for *M*. *smegmatis*

Extracts were prepared as described [56] with minor modifications. 200 ml Middlebrook 7H9 medium was inoculated with 2.0 ml of an overnight *M*. *smegmatis* culture, and grown at 37°C to an OD_600_ of ~1.0. Chloramphenicol was added to 100 μg/ml 2 minutes before harvesting cells to stabilize the mRNA-associated ribosomes. Cells were harvested by rapid filtration using a 500 ml 0.45 μm PES filter system (Celltreat) and flash frozen in liquid nitrogen together with 0.7 ml lysis buffer [56]. The frozen cells were pulverized 6 times at 15 Hz for 3 min in a mixer mill (Retsch MM400). The grinding jars were re-chilled in liquid nitrogen to keep the cells frozen between each cycle. After the pulverized cells were recovered, a small aliquot was saved to assess bacterial transcript enrichment.

### Transcript enrichment for RNA-seq

The pulverized cell powder was extracted with acid phenol and chloroform followed by isopropanol precipitation [56]. 16S and 23S ribosomal RNAs were removed by subtractive hybridization using a Ribo-Zero Magnetic kit (Epicentre) following the manufacturer’s protocol. The enriched mRNAs were randomly fragmented as described [56].

### Ribosome profiling of mRNA

Ribosome profiling was performed as described [56]. Briefly, the pulverized cells were thawed and the soluble cytoplasmic fraction isolated by centrifugation of insoluble material. The clarified lysates were treated with micrococcal nuclease to degrade DNA and reduce polysomes to monosomes (MNase, Worthington Biochemical Corp). Monosomes were isolated by sucrose-gradient fractionation. mRNA was extracted from the monosome fraction by treatment with acid phenol and chloroform extraction, and isopropanol precipitation.

### Library generation and deep sequencing

Both ribosomal footprints and the enriched mRNAs were converted into cDNA libraries as described [56, 57] with minor modifications. The RNA molecules were dephosphorylated by treatment with T4 polynucleotide kinase (New England Biolabs). Then polyacrylamide gel purification was performed for size selection of ~28 nt RNA fragments. Approximately 25–30 nt poly-A tails were added to recovered RNA fragments with an Ambion poly(A) tailing kit (Life Technologies) following the manufacturer’s protocol. The polyadenylated RNA samples were reverse transcribed using SuperScript III (Life Technologies), and primer JW2364 (/5Phos/GATCGTCGGACTGTAGAACTCTGAACCTGTCGGTGGTCGCCGTATCATT/iSp18/CACTCA/iSp18/CAAGCAGAAGACGGCATACGATTTTTTTTTTTTTTTTTTTTVN)[57]. The reverse transcription products were circularized by CircLigase ssDNA ligase (Epicentre). For the footprint libraries, ribosomal RNAs were subtracted from circularized ssDNA using biotinylated sense-strand oligonucleotides JW4267 (/5Biosg/TATCCTGAGAGGTGATGCATAGCCG), JW4268 (/5Biosg/TAAACGGTGGGTACTAGGTGTGGGTTTC), JW4269 (/5Biosg/CTTGGGATCCGTGCCGTAGCTAACGCAT), JW4270 (/5Biosg/AGGAAGGTAGCCGTACCGGTCAGTG), and JW4271 (/5Biosg/CACACCGCCGAAGCCGCGGCAGCCAAC). PCR amplification was performed using the circularized cDNA as template, JW2365 AATGATACGGCGACCACCGA) and JW2366 (CAAGCAGAAGACGGCATACGA) as primers[57], and Phusion High-Fidelity DNA Polymerase (New England Biolabs). The PCR amplified DNA libraries were deep sequenced by Illumina HiSeq 2500 (University at Buffalo Next-Generation Sequencing and Analysis Expression Core Facility). Sequence reads were mapped to the mc^2^155 reference sequence (GenBank CP000480.1).

Raw read counts for both RNAseq and RiboSeq replicates (S7 Fig) were added and merged together prior to prediction of TSS. TSS were predicted using adjacent sliding windows of multiple sizes and by calculating the ratio of total-read counts between the windows (Downstream window counts / Upstream window counts). The windows were moved one nucleotide at a time along the reference genomes. The window sizes used for ratio calculations were of 10 nt, 15 nt, 25 nt, 35 nt, 50 nt, 75 nt, 100 nt, 125 nt, 150 nt, 200 nt, 250 nt, 300 nt, and 350 nt. Smaller window sizes allow detection of sharp increases of reads counts along the data, while larger windows are more suitable for detecting shallower but constant rate increases. The window ratios at each position along the genome were assessed to determine the presence of a boundary (or peaks) marking the onset of transcription (Bioinformatics & Statistics Core, Wadsworth Center). A potential transcription start site was defined as the position in a genome of the maximum height of a peak equal >50 for the RNA-seq data and >100 for the ribosome profiling data. Leaderless TSS were determined by looking at RNAseq or ribosome profiling boundaries that fell directly at RTG positions in the genome. The list of boundaries were compared to the annotations provided by the J. Craig Venter Institute (JCVI) to identify genes that were correctly or incorrectly annotated using traditional annotation tools.

To reduce the false-positive rate with any single dataset, independent datasets were often combined to generate a working list of high-confidence calls. The high-confidence leaderless transcript dataset in *M*. *smegmatis* was generated from the overlap of RTGs at RNA-seq boundaries (633) and ribosome profiling boundaries (1362) for 498 total. Mapped reads from RNA-seq and ribosome profiling were also visualized in a SignalMap browser (Nimblegen), with annotations provided by the J. Craig Venter Institute (JCVI) and PathoSystems Resource Integration Center (PATRIC).

### Transcription start site mapping in *M*. *tuberculosis*

Biological replicate cultures were grown to an optical density of 1 in roller bottles and inactivated with RNAlater (Ambion) prior to pelleting and RNA extraction. Pellets from 18 ml of culture each disrupted in 1 mL Trizol (Life Technologies) in Lysing Matrix B tubes (MP) in a FastPrep-24 (MP) using two cycles of 30–45 seconds at 6.5 m/sec. 300 μL chloroform was added, samples were centrifuged for 15 minutes at 4°C, supernatants were mixed with equal volumes of isopropanol, and the resulting samples were incubated for 1 hour at -20°C before centrifugation for 10 minutes at 4°C. Pellets were washed with 75% ethanol, resuspended in water, treated with DNase Turbo (Ambion) and purified by RNeasy (Qiagen) as directed by the manufacturer. RNA samples were subject to two consecutive rounds of ribosomal RNA depletion using a MICROBExpress kit (Ambion) as directed by the manufacturer.

Transcription start sites were mapped as described in [58] with modifications. Briefly: RNA samples from two biological replicates were each processed to create two parallel libraries: a “converted” library, which captured RNA 5’ ends bearing 5’ triphosphates and 5’ monophosphates, and a “non-converted” library, which captured only RNA 5’ ends bearing 5’ monophosphates. The resulting libraries bore Illumina TruSeq adapter sequences, with the first base of Read 1 corresponding to the exact 5’ end of an RNA molecule. To convert triphosphates to monophosphates in the “converted” library, 1.7 μg rRNA-depleted RNA was incubated with 1 μL 5’ Polyphosphatase (Epicentre) in a 20 μL reaction for 1 hour at 37°C. For the “non-converted” library, 1.7 μg rRNA-depleted RNA was incubated in parallel in a mock reaction lacking enzyme. Samples were purified by RNeasy (Qiagen) and eluted in 100 μL water containing RNaseOUT (Life Technologies) and DTT. One μL 100 mM Tris pH 7.5 was added and samples were concentrated to 8 μL by vacuum centrifugation. Concentrated samples were mixed with 1 μL of 5 μg/μL oligo SSS392 (TCCCTACACGACGCTCTTCC*GAUCU*; normal font indicates deoxyribonucleotides and italics indicate ribonucleotides), denatured at 65°C. for 10 minutes, and cooled in an ice-water bath. Three μL 10X T4 RNA ligase buffer (New England Biolabs), 1 μL RNaseOUT, 10 μL 50% PEG8000, 3 μL RNaseOUT, 10 μL of 10 mM ATP, 3 μL DMSO, and 1 μL T4 RNA ligase I (New England Biolabs) were added and reactions incubated 18 hours at 20°C. Seventy μL water was added and samples were purified by RNeasy (Qiagen) and eluted in 126 μL water. Samples were sheared in a Covaris sonicator in AFA MicroTubes (Covaris) as follows: exposure time, 180 seconds; duty cycle, 10%; intensity, 5; cycles/burst, 200. One μL each Tris pH 7.5, 100 mM DTT, and RNaseOUT (Life Technologies) were added and samples concentrated to 11.25 μL. One μL of 2.2 μg/μL SSS397 (CTGGAGTTCAGACGTGTGCTCTTCCGATCTNNNNNN, where “N” represents a degenerate base) was added, samples were incubated at 65°C for 10 minutes and cooled in an ice-water bath. Four μL 5X first-strand buffer (Life Technologies), 1 μL dNTP mix containing 10 mM each dNTP, 0.5 μL RNaseOUT (Life Technologies), 0.25 μL of 1 mg/mL actinomycin D, 1 μL 100 mM DTT, and 1 μL Superscript III (Life Technologies) were added and reactions incubated 18 hours at 42°C. RNA was degraded by treatment with 10 μL each 1 N NaOH and 500 mM EDTA at 65°C. for 15 minutes, 25 μL 1 M Tris pH 7.5 was added, and cDNA was purified by MinElute kit (Qiagen) and eluted in 60 μL water.

To add the remaining adapter sequences, samples were subject to PCR as follows: 10 μL 5X HF buffer (Finnzymes), 2.5 μL of 10 μM oligo SSS398 (AATGATACGGCGACCACCGAGATCTACACTCTTTCCCTACACGACGCTCTTC), 2.5 μL of 10 μM reverse oligo (CAAGCAGAAGACGGCATACGAGATXXXXXXGTGACTGGAGTTCAGACGTGTGCT, where “XXXXXX” is a 6 nt Illumina index sequence), 20 μL 5 M betaine, 0.5 μL Phusion polymerase (Finnzymes), 0.4 μL dNTP mix containing 25 mM each dNTP, and 14.1 μL purified cDNA from the previous step. Thermal cycler settings: 1 cycle of 98°C for 3 minutes, 8 cycles of 98°C for 80 seconds, 60°C for 30 seconds, and 72°C for 30 seconds, 1 cycle of 72°C for 5 minutes. Reaction volumes were set to 100 μL on the thermal cycler to increase ramp time [59]. Three reactions were performed for each sample. Products between 150 and 500 nt were size-selected on a 1.5% agarose gel, purified by a Qiagen gel extraction kit, eluted in 40 μL water, and replicates were pooled and concentrated by vacuum centrifugation to a final volume of 50 μL per library. Samples were further purified by Ampure XP beads (Agencourt) using a beads:sample ratio of 1.8 and an elution of 50 μL water. To enrich for molecules with full-length adatpers, products were subject to an additional PCR as follows: 10 μL 5X HF buffer (Finnzymes), 2.5 μL of 10 μM oligo SSS401 (AATGATACGGCGACCACCGAGATC), 2.5 μL of 10 μM oligo SSS402 (CAAGCAGAAGACGGCATACGAGAT), 20 μL 5 M betaine, 0.5 μL Phusion polymerase (Finnzymes), 0.4 μL dNTP mix containing 25 mM each dNTP, and 14.1 μL purified PCR product from the previous step. Thermal cycler settings: 1 cycle of 98°C for 3 minutes, 4 cycles of 98°C for 80 seconds, 60°C for 30 seconds, and 72°C for 30 seconds, 1 cycle of 72°C for 5 minutes. Reaction volumes were set to 100 μL on the thermal cycler to increase ramp time [59].

Three reactions were performed for each sample. Products were purified twice using Ampure XP beads (Agencourt) with a beads:sample ratio of 1.8 and an elution of 60 μL water.

RNA-seq expression libraries were made from the same samples used for 5’-end mapping. 1 μg of MICROBExpress-treated RNA from each biological replicate was sheared by a Covaris sonicator as follows: exposure time, 180 seconds; duty cycle, 10%; intensity, 5; cycles/burst, 200. Samples were concentrated by vacuum centrifugation and cDNA was synthesized by adding 3 μg of random primers (Life Technologies), incubating at 70°C for 10 minutes and snap-cooling in an ice water bath, adding 4 μL 5X first-strand buffer (Life Technologies), 1 μL dNTP mix containing 10 mM each dNTP, 0.5 μL RNaseOUT (Life Technologies), 0.5 μL of 1 mg/mL actinomycin D, 1 μL 100 mM DTT, and 1 μL Superscript III (Life Technologies) and incubating overnight at 42°C. RNA was degraded and cDNA purified as described above for 5’ end mapping libraries. Each first-strand cDNA sample was divided into 8 replicate second-strand synthesis reactions. Approximately 100 ng cDNA was mixed with 3 μg of random primers (Life Technologies) and 2 μL 100 mM Tris pH 7.5, incubated at 95°C for 3 minutes, cooled rapidly to 50°C, and then cooled from 50°C to 4°C at a rate of -0.4°C/second. The following were added before an overnight incubation at 16°C: 8 μL NEBNext Second Strand Synthesis (dNTP-free) Reaction Buffer, 1.6 μL dNTP mix containing 10 mM each dATP, dCTP, dGTP, and dUTP, 4 μL NEBNext Second Strand Synthesis enzyme mix, and water to a final volume of 80 μL. Reactions were purified with MinElute columns (Qiagen). 1 μg of purified second-strand synthesis reaction was then subject to end repair, A-tailing, and adapter ligation using a TruSeq DNA Sample Preparation Kit (Illumina) according to the manufacturer’s instructions. Following the ligation and subsequent clean-up steps, the entire product was run on a 2% agarose gel and the region between approximately 250 and 600 bp was excised and extracted with a Qiagen gel extraction kit. Each sample was divided into 4 reactions containing approximately 80 ng DNA, 1 μL 100 mM Tris pH 7.5, and 2 μL USER enzyme mix (NEB) and incubated at 37°C for 30 minutes. The following were added for PCR: 10 μL 5X HF buffer (Finnzymes), 5 μL primer mix from Illumina TruSeq kit, 0.4 μL dNTP mix that contains 25 mM each dNTP, 20 μL 5 M betaine, and 0.5 μL Phusion polymerase (Finnzymes). Thermal cycler conditions were as follows: 1 cycle of 98°C. for 3 minutes, 10 cycles of 98°C for 1 minute, 60°C for 30 seconds, and 72°C for 30 seconds, 1 cycle of 72°C for 5 minutes. Reaction volumes were set to 100 μL on the thermal cycler to increase ramp time [59].

Duplicate reactions were combined and purified twice with Ampure XP beads (Agencourt) according to the manufacturer’s instructions. Libraries were sequenced on an Illumina Genome Analyzer in a paired-end run producing 56 nt reads.

Ssaha2 was used to map reads to *M*. *tuberculosis* H37Rv genome version NC_000962. The 1^st^ base of “Read 1” (hereafter called “1^st^ nt”) was extracted to determine the number of RNA 5’ ends mapped to each genome coordinate. Zero values were replaced by 0.5. To identify above-background peaks in 5’ end coverage, the mean coverage at each coordinate in the two biological replicate “converted” libraries was averaged and the ratios of “1^st^ nt” coverage at each coordinate relative to the positions 10 nt upstream and 10 nt downstream were determined. Approximately 22,000 peaks were identified where at least one of the two ratios was over 25. These peaks in 5’ end coverage were subject to the following filters: (1) peaks with a mean “converted” library coverage under 20 were removed; and (2) the ratio of mean “converted” library “1^st^ nt” coverage to mean RNA-seq expression library coverage in the 200 nt region upstream of the “1^st^ nt” peak was determined, and only peaks with ratios of at least 0.7 were retained.

For each filtered RNA 5’ end, the “1^st^ nt” coverage in the replicate libraries was summed and the ratio in the converted/non-converted libraries was determined. The distribution of ratios was bimodal, and Gaussian mixture modeling was used to estimate the means and standard deviations for two skewed normal distributions (one comprised of processed 5’ ends, and one comprised of unprocessed 5’ ends corresponding to transcription start sites). RNA 5' ends with ratios greater than 1.74 had a cumulative probability of ≤0.01 of belonging to the processed 5’ end population (after adjusting for multiple comparisons by the Benjamini-Hochberg procedure) and were therefore designated transcription start sites (TSSs). Because transcriptional initiation is imprecise, the 6,900 statistically significant TSSs were filtered to remove all but the single TSS with the highest converted-library coverage in each 11 nt window. 4,978 TSSs passed this filter and are included in S3 Table.

### Proteomic analyses

Whole-cell protein lysates were prepared as described in [55]. Pelleted lysates were resuspended in 6 M urea/50 mM ammonium bicarbonate. Protein content of each sample was measured using Pierce BCA protein assay. 20 mM DTT was added to 500 μg of protein and samples were incubated for 30 minutes at 37°C. Iodoacetamide was added at a final concentration of 50 mM and samples were incubated for 30 minutes in the dark at room temperature. Prior to trypsin digestion, urea concentration was diluted to less than 1 M by adding water and pH adjusted to 8 with a 1 M Tris solution. 10 μg sequencing grade trypsin (Cat. No. V5280, Promega, Madison, WI) was added (1:50 enzyme to substrate ratio) and samples were incubated at 37°C with shaking for 16 hours. The reaction was stopped by addition of formic acid (FA) to a final concentration of 1% and the solution was desalted with a 1 cc (30 mg) Oasis HLB reverse phase cartridge (Cat. No. WAT054955, Waters, Milford, USA) conditioned with 3 x 500 μL acetonitrile (ACN), followed by 4 x 500 μL 0.1% FA. Samples were loaded onto the cartridges and washed with 3 x 500 μL 0.1% FA. Desalted peptides were eluted by 2 applications of 500 μL of 80% ACN/0.1% FA. Eluates were frozen, dried via vacuum centrifugation prior to peptide fractionation. The sample was then fractionated by reverse phase chromatography into 24 fractions and analyzed by liquid chromatography—tandem mass spectrometry. Briefly, each of the 24 fractions were resuspended in 20 μL of 3% ACN/ 0.5% FA and 2 μL of the peptide mixture was injected and separated by a 100 min gradient (~0.7%B/min.) of increasing acetonitrile from 5–60%B. A PicoFrit column (New Objective, Woburn, MA), with an inner diameter of 75 μm packed with 12–14 cm of ReproSil-Pur C18 3 μm particles, was directly interfaced to an Agilent 1100 HPLC coupled Orbitrap Velos mass spectrometer (Thermo) equipped with a custom nano-electrospray ionization source. MS analysis settings for protein identification were as follows. One precursor MS scan at 60,000 resolution in profile mode was followed by data-dependent scans of the top 15 most abundant ions at low-resolution in centroid mode. Dynamic exclusion was enabled with a repeat count of 2, repeat duration of 20 seconds, exclusion duration of 30 seconds and an exclusion list size of 500. MS/MS spectra were collected with normalized collision energy of 28 and an isolation width of 3 amu. The extensive peptide fractionation coupled with in depth MS analysis allowed detection and identification of very low levels of peptides. All MS data were processed using Agilent Spectrum Mill MS Proteomics Workbench (Agilent Technologies, Palo Alto, USA).

### Directed β-galactosidase assays

Specific codons were tested for their ability to initiate translation of the *lacZ* gene by generating translational fusions. Briefly, oligonucleotides were synthesized with candidate initiation codons (ATG, CTG, GTG, or TTG = NTG) at either the leadered or leaderless positions. A well-characterized *E*. *coli* derived promoter with a mapped +1 transcription start site was used to drive expression [60]. Putative negative control codons (NTC) were also substituted to evaluate specificity and robustness of translation initiation at that codon. Since altering the +1 nucleotide could conceivably alter transcriptional robustness—as reported for T7 RNA polymerase [49]—substituting this nucleotide in the conventional leadered reporters controlled for this potential variable. Overlapping oligonucleotides were paired to create the desired leaderless- and leadered-codon combinations, extended to duplex DNAs by self-templating PCR, and cloned into the *lacZ* reporter using 15-nt recombination arms in the promoter region and *lacZ* ORF by InFusion (Clontech). *E*. *coli* cultures were grown in LB supplemented with hygromycin, and *M*. *smegmatis* in 7H9 supplemented with Tween 80 and hygromycin. Cultures were expanded, their density determined by OD_600_, and lysed crude extracts were prepared and β-galactosidase activities measured by ONPG conversion and reading at OD_420_. Activities were calculated in Miller Units, and displayed as percentages relative to a leadered ATG control construct.

### Zeo-seq viability translation initiation assays

A zeocin-resistance gene reporter was created to allow expression by translational fusion, as for the *lacZ* reporter. The same basic oligonucleotide design described for *lacZ* was modified to accommodate the *zeo* ^*r*^ gene overlap for InFusion cloning. Clusters of nucleotides of each oligo were specified for functionality (e.g., ATG or ATC codons, Shine-Dalgarno sequence) or unspecified with random nucleotides to allow subsequent selection. Duplex DNAs were created by a single round of bidirectional primer extension and the library was created by InFusion cloning. Hygromycin-resistant *E*. *coli* colonies (>10,000 per library pool) were collected and a plasmid library was created for electroporation into *M*. *smegmatis*. Hygromycin-resistant *M*. *smegmatis* colonies (>8,000 colonies) were scraped and grown under zeocin (100 μg/ml) selection in TSA + 0.05% Tween 80. Plasmids were purified with aliquots from the Hyg-resistant and Zeo-resistant pools, and these were used as templates for PCR amplification of the cloned leader segments for amplicon-based next generation sequencing (Ion Torrent). Equivalent amounts of the barcoded amplicons were pooled and sequenced on an Ion 318 chip (Applied Genomic Technologies Core, Wadsworth Center). Sequence reads were aligned, trimmed, and the sequences at the randomized positions were compiled. Recovered sequences are shown as log_10_ converted heat maps of every possible codon, or as sequence logos for supporting elements. The enrichment of all codon combinations that contain at least one active initiation codon, with the notable exception of stop codons at the leadered position, indicates that clonal jackpot artifacts are not a major problem.

### Coupled small ORFs analysis

Two hundred and twenty leaderless (begin with an RTG) *M*. *tuberculosis* transcripts were identified that initiate small ORFs of 5–50 amino acids. The stop codons of these small ORFs were mapped relative to the annotated start of the next downstream ORF, and 23 were found to have coupled architectures (RTGA) at the junction of the two ORFs. To determine whether 23 of 220 represented an enriched population, 1000 random sites in the genome were considered arbitrary start sites for ORFs that were similarly analyzed. Of the 1000 randomly identified *in silico* ORFs, 32 were coupled to a downstream ORF. The enrichment of 23/220 to 32/1000 was determined to be significant (p < 0.0003, one-tailed Fisher’s Exact test).

### Data sharing

The primary sequence data have been deposited in public repositories. The *M*. *smegmatis* RNA-seq and Ribo-seq data can be found at the European Nucleotide Archive <https://www.ebi.ac.uk/arrayexpress/experiments/E-MTAB-2929/>. The *M*. *tuberculosis* RNA-seq and the TSS data have been submitted to GEO under the accession number GSE62152. The Mass Spec data used for N-terminal peptide identification have been submitted to MassIVE, ID number MSV000079012, password 1tuberculosis. The data are also posted in the form of mapped sequence reads (Applied Genomic Technologies Core, Wadsworth Center), and viewed at http://www.wadsworth.org/research/scientific-resources/interactive-genomics/.

## Supporting Information

S1 Fig Leaderless ATG or GTG (RTG) codons define sites of translation initiation. (A) Leaderless RTGs often initiate an annotated ORF at the annotated initiation codon. The RTG codon may specify an in-frame, upstream start that will add residues (gray) to the N-terminus of the predicted protein. Alternatively, it may initiate downstream of the annotated start codon and will omit residues from the predicted N-terminus. The annotated stop codon is unchanged. (B) RTG codons not initiating an annotated reading specify novel, unannotated, ORFs. These novel ORFs represent the first gene of this leaderless operon transcript, and frequently predict small proteins (sp) of under 50 amino acids. These novel ORFs terminate upstream of the predicted annotated start downstream (uORF), or they overlap in the -1 frame via a coupled tetramer, or extend into the annotated gene, utilizing a different frame.
(PDF)

S2 Fig Distribution of RTG codon distance from 5’ ribosome footprint boundaries in *M*. *smegmatis*. Enrichments of RTG codons were found at the boundary (leaderless) and in the distances surrounding 24 nt of separation between the leading edge of the footprint and a candidate RTG codon (leadered). The observed twin peaks profile would be expected from populations of 70S ribosomes poised over either leaderless or leadered translation initiation RTG codons.
(PDF)

S3 Fig Shine-Dalgarno-like sequences identified near candidate leadered initiation codons from *M*. *smegmatis* transcripts. The 19 nt upstream of 731 candidate RTG codons 20–30 nt from a ribosome footprint boundary were analyzed for sub-sequence enrichment <http://meme.nbcr.net/meme/tools/meme>. The two motifs with significant scores are pictured. Therefore, approximately one-half of the candidate leadered RTGs identified by ribosome profiling have an upstream sequence resembling a consensus Shine-Dalgarno sequence (AGGAGG), with the upper motif conforming to the Shine-Dalgarno core, and the lower motif representing a possible degenerate Shine-Dalgarno. The remaining candidate 5’ UTRs either had sequences that were too diverged from the canonical SD-like core element shared by the group above for inclusion, or they represent RNA fragments where the candidate initiating RTG is not a codon, or is internal to the transcript and is not a site of initiation.
(PDF)

S4 Fig Rv2122c and MSMEG_4181 exemplify phylogenetically corroborated mis-annotation. Rv2122c and Msmeg_4181 provide an example of phylogenetically corroborated mis-annotation. (A) The *M*. *tuberculosis* gene, Rv2122c, has an empirically determined transcription start site 18 nucleotides into the annotated open reading frame. (B) The *M*. *smegmatis* ortholog of Rv2122c, Msmeg_4181, also has RNA-seq and ribosome footprint boundaries beginning 18 nucleotides into the same predicted open reading frame. (C) Nucleotide alignment of the annotated ORFs with 20 additional nucleotides of 5’ sequence. The green outlined box frames the JCVI annotated initiation codon for which there is no supporting evidence (note that the ORF predicted by PATRIC for *M*. *smegmatis* is an ATG at +12). TSS, RNA-seq, and ribosome footprinting data are all consistent with transcription and translation initiating with the green shaded GTG codon at +18. The conserved -10 promoter nucleotides and the preferred -1 cytosine are shaded in blue. The TGA stop codon (unchanged) is in a red-shaded box.
(PDF)

S5 Fig Near-leaderless RTG distribution and translation efficiency as supported by N-terminal peptide mapping. Potential initiating leaderless RTG codons were mapped by position relative to the transcription start site (TSS) in M. tuberculosis (dark bar, left axis). The distribution of RTGs is heavily weighted to the +1 site (left most bar). The number of N-termini that map to each position is also shown (red bar, right axis). The percentage of RTGs that are experimentally supported (number of N-terminal peptides per 100 RTGs at that position) is shown above each N-terminal tally.
(PDF)

S6 Fig Coupling of annotated genes within putative operons in M. smegmatis. Both upstream and downstream ORFs showed the expected GC codon bias. The wobble positions of the upstream gene reflect the Shine-Dalgarno consensus sequence (black box), suggesting the coexistence of coupling and canonical translation initiation at these junctions.
(PDF)

S7 Fig Duplicate dataset plots for RNA-seq and ribosome footprinting in *M*. *smegmatis*. *M*. *smegmatis* transcriptome datasets were generated in duplicate for both the RNA-seq and ribosome profiling. Compiled read counts from genes in each dataset plotted on log axes show the highly correlative line of identity typical of genome scale studies. Read counts for a given gene in each experiment were normalized for gene length (average reads per nucleotide mapped along the gene), converted to log scale, and plotted as shown. Poorly expressed genes averaging a read depth of less than 1 have negative log conversions. RNA-seq (A) and ribosome profiling (B) replicates are shown.
(PDF)

S1 Table *M*. *smegmatis* leaderless genes. (XLSX)

S2 Table Ribosome profiling fragment 5’ boundaries in *M*. *smegmatis*. (XLSX)

S3 Table Transcription start sites in *M*. *tuberculosis*. (XLSX)

S4 Table Protein N-termini in *M*. *tuberculosis*. (XLSX)

S5 Table Zeo-seq sequences. (XLSX)

S6 Table Sequence and coordinates of all small proteins. (XLSX)
