# Supplementary material for: Leaderless Transcripts and Small Proteins Are Common Features of the Mycobacterial Translational Landscape
Source: PLoS Genet. 2015 Nov 4;11(11):e1005641. doi: 10.1371/journal.pgen.1005641 (PMC4633059; doi:10.1371/journal.pgen.1005641)

Supporting Information Figure S1

## (A) RTG codon initiates in annotated frame

same or new N-terminus of annotated protein, but C-terminus is unchanged.

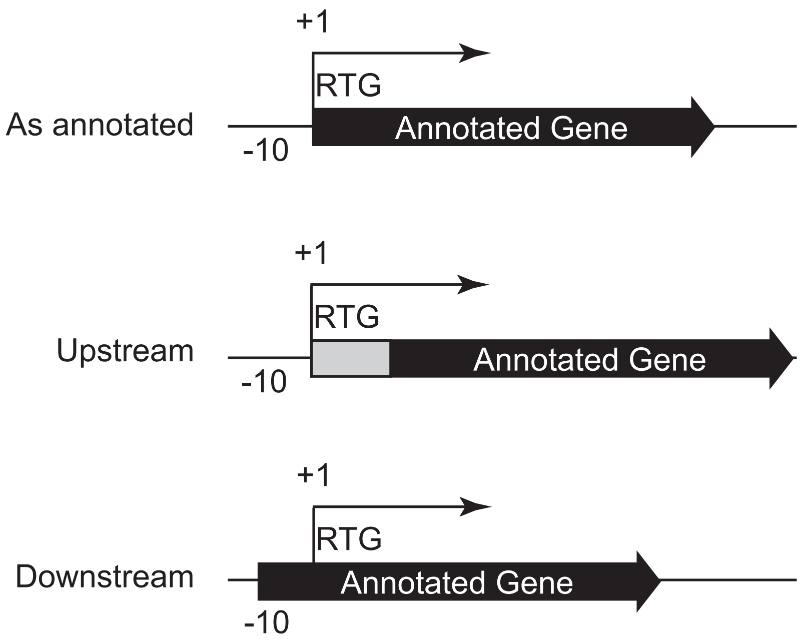


## (B) RTG codon initiates novel 5' ORF

three ORF termination classes relative to annotated dowsntream gene.

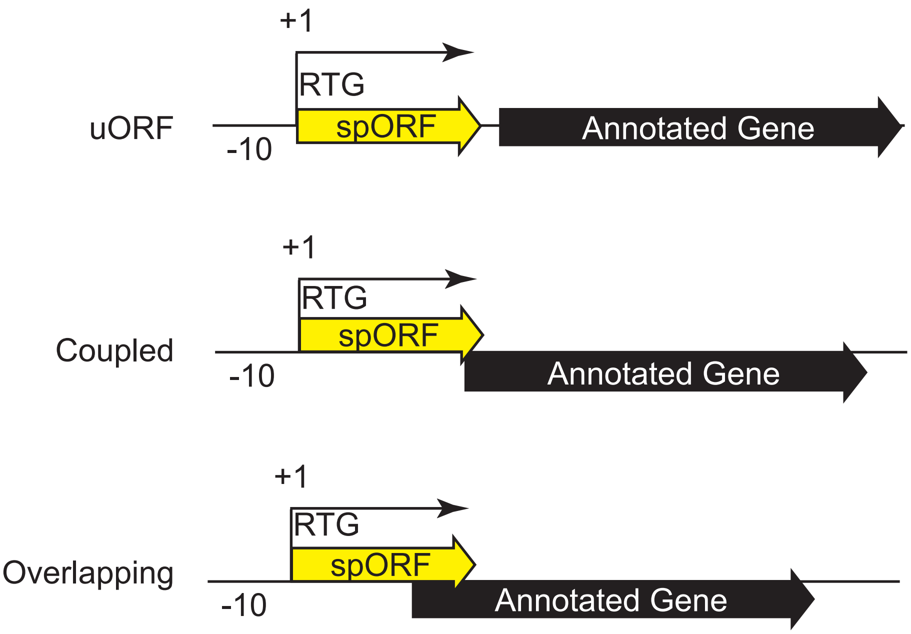

Supplement: S1 Fig — (A) Leaderless RTGs often initiate an annotated ORF at the annotated initiation codon. The RTG codon may specify an in-frame, upstream start that will add residues (gray) to the N-terminus of the predicted protein. Alternatively, it may initiate downstream of the annotated start codon and will omit residues from the predicted N-terminus. The annotated stop codon is unchanged. (B) RTG codons not initiating an annotated reading specify novel, unannotated, ORFs. These novel ORFs represent the first gene of this leaderless operon transcript, and frequently predict small proteins (sp) of under 50 amino acids. These novel ORFs terminate upstream of the predicted annotated start downstream (uORF), or they overlap in the -1 frame via a coupled tetramer, or extend into the annotated gene, utilizing a different frame. (PDF) [file pgen.1005641.s001.pdf]
