# Supplementary figures and images for: Leaderless Transcripts and Small Proteins Are Common Features of the Mycobacterial Translational Landscape
Source: PLoS Genet. 2015 Nov 4;11(11):e1005641. doi: 10.1371/journal.pgen.1005641 (PMC4633059; doi:10.1371/journal.pgen.1005641)

Supporting Information Figure S2

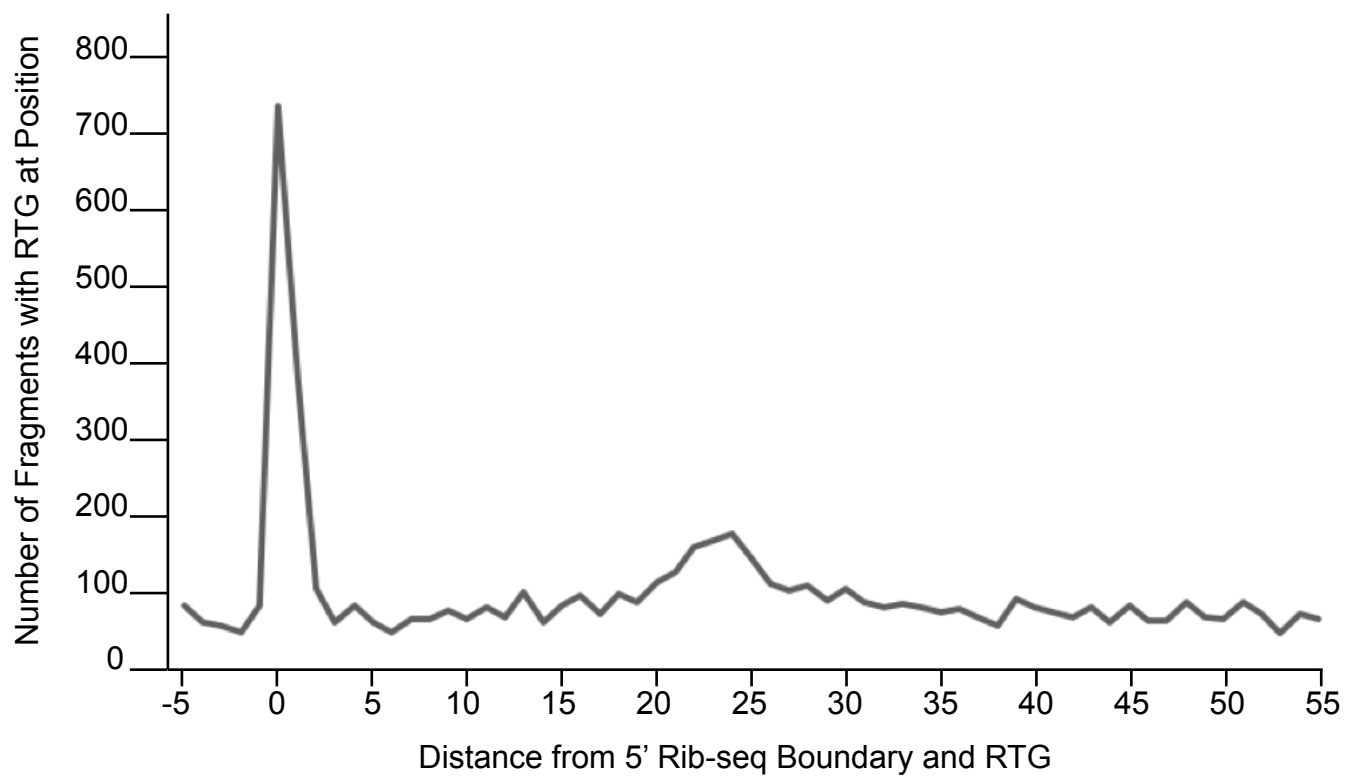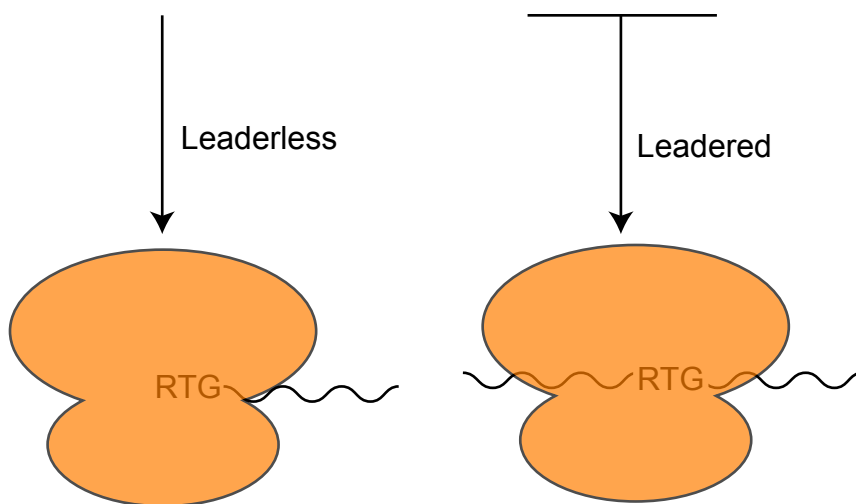

Supplement: S2 Fig — Enrichments of RTG codons were found at the boundary (leaderless) and in the distances surrounding 24 nt of separation between the leading edge of the footprint and a candidate RTG codon (leadered). The observed twin peaks profile would be expected from populations of 70S ribosomes poised over either leaderless or leadered translation initiation RTG codons. (PDF) [file pgen.1005641.s002.pdf]

Supporting Information Figure S3

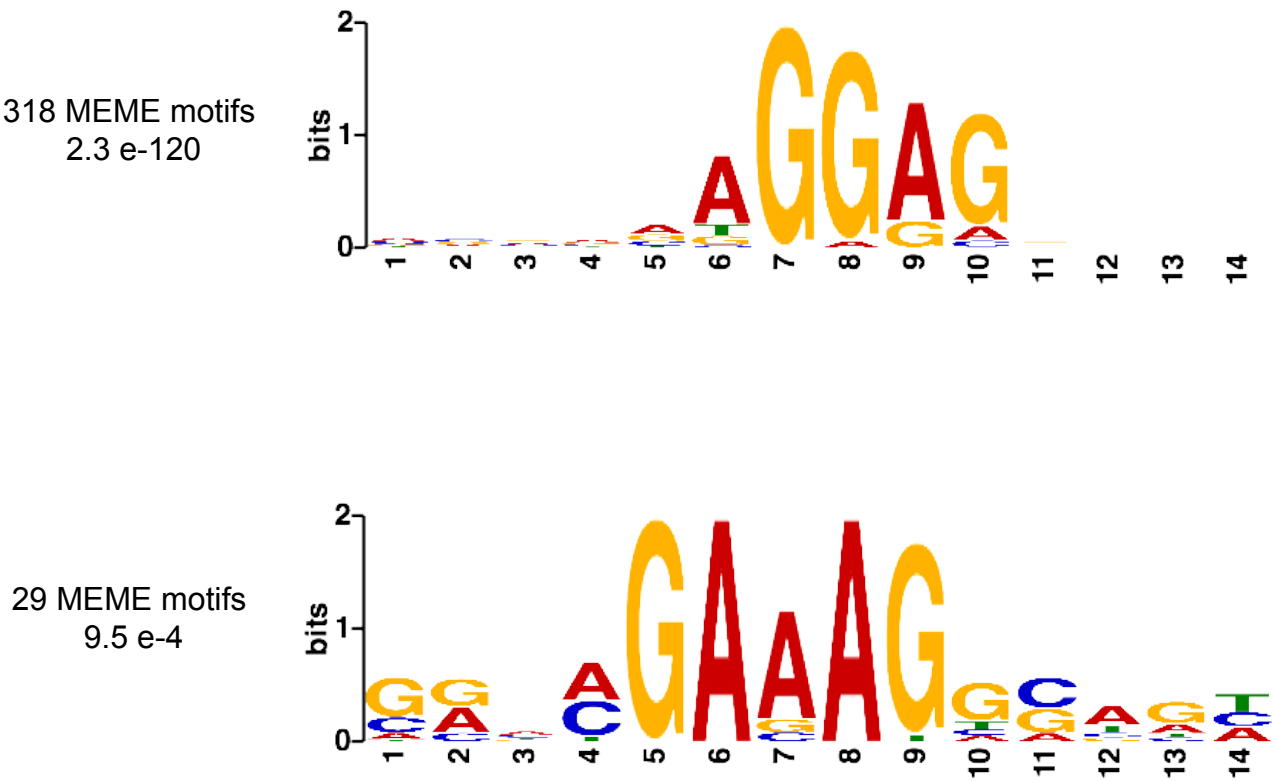

Supplement: S3 Fig — The 19 nt upstream of 731 candidate RTG codons 20–30 nt from a ribosome footprint boundary were analyzed for sub-sequence enrichment <http://meme.nbcr.net/meme/tools/meme>. The two motifs with significant scores are pictured. Therefore, approximately one-half of the candidate leadered RTGs identified by ribosome profiling have an upstream sequence resembling a consensus Shine-Dalgarno sequence (AGGAGG), with the upper motif conforming to the Shine-Dalgarno core, and the lower motif representing a possible degenerate Shine-Dalgarno. The remaining candidate 5’ UTRs either had sequences that were too diverged from the canonical SD-like core element shared by the group above for inclusion, or they represent RNA fragments where the candidate initiating RTG is not a codon, or is internal to the transcript and is not a site of initiation. (PDF) [file pgen.1005641.s003.pdf]

Supporting Information Figure S4

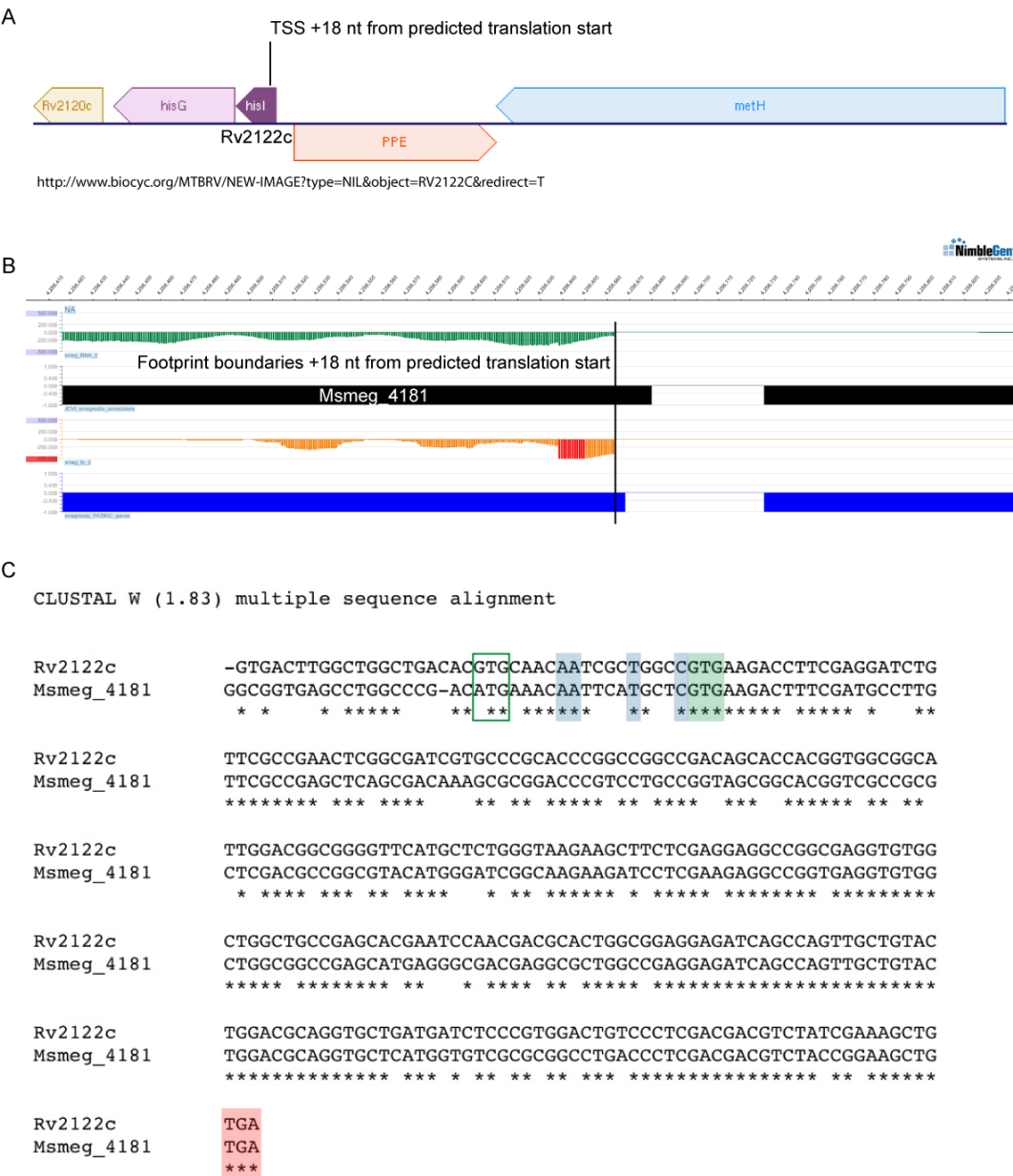

Supplement: S4 Fig — Rv2122c and Msmeg_4181 provide an example of phylogenetically corroborated mis-annotation. (A) The M. tuberculosis gene, Rv2122c, has an empirically determined transcription start site 18 nucleotides into the annotated open reading frame. (B) The M. smegmatis ortholog of Rv2122c, Msmeg_4181, also has RNA-seq and ribosome footprint boundaries beginning 18 nucleotides into the same predicted open reading frame. (C) Nucleotide alignment of the annotated ORFs with 20 additional nucleotides of 5’ sequence. The green outlined box frames the JCVI annotated initiation codon for which there is no supporting evidence (note that the ORF predicted by PATRIC for M. smegmatis is an ATG at +12). TSS, RNA-seq, and ribosome footprinting data are all consistent with transcription and translation initiating with the green shaded GTG codon at +18. The conserved -10 promoter nucleotides and the preferred -1 cytosine are shaded in blue. The TGA stop codon (unchanged) is in a red-shaded box. (PDF) [file pgen.1005641.s004.pdf]

Supporting Information Figure S5

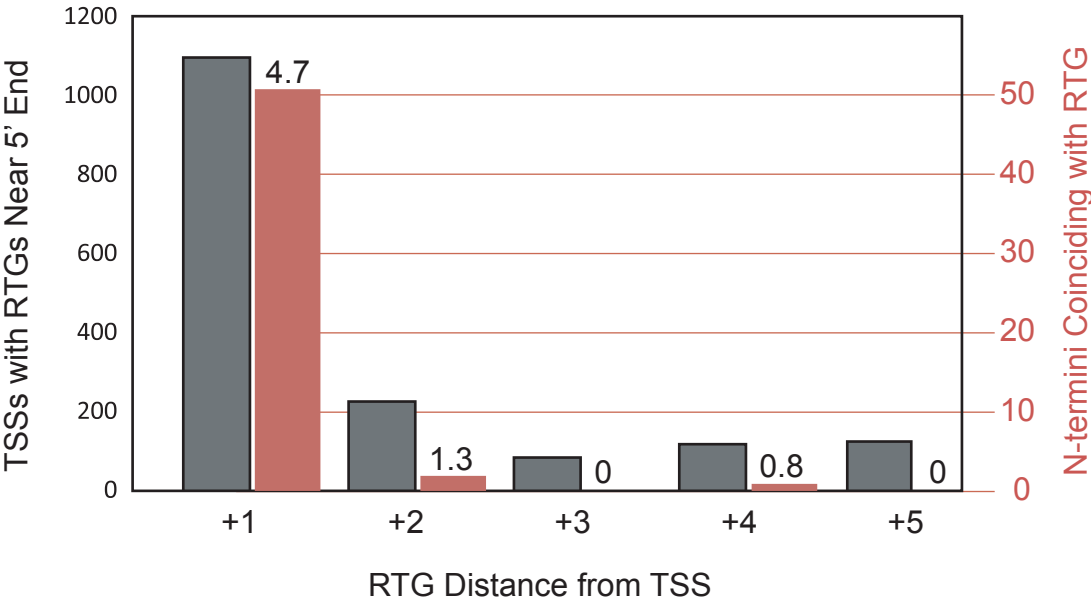

Supplement: S5 Fig — Potential initiating leaderless RTG codons were mapped by position relative to the transcription start site (TSS) in M. tuberculosis (dark bar, left axis). The distribution of RTGs is heavily weighted to the +1 site (left most bar). The number of N-termini that map to each position is also shown (red bar, right axis). The percentage of RTGs that are experimentally supported (number of N-terminal peptides per 100 RTGs at that position) is shown above each N-terminal tally. (PDF) [file pgen.1005641.s005.pdf]

Supporting Information Figure S6

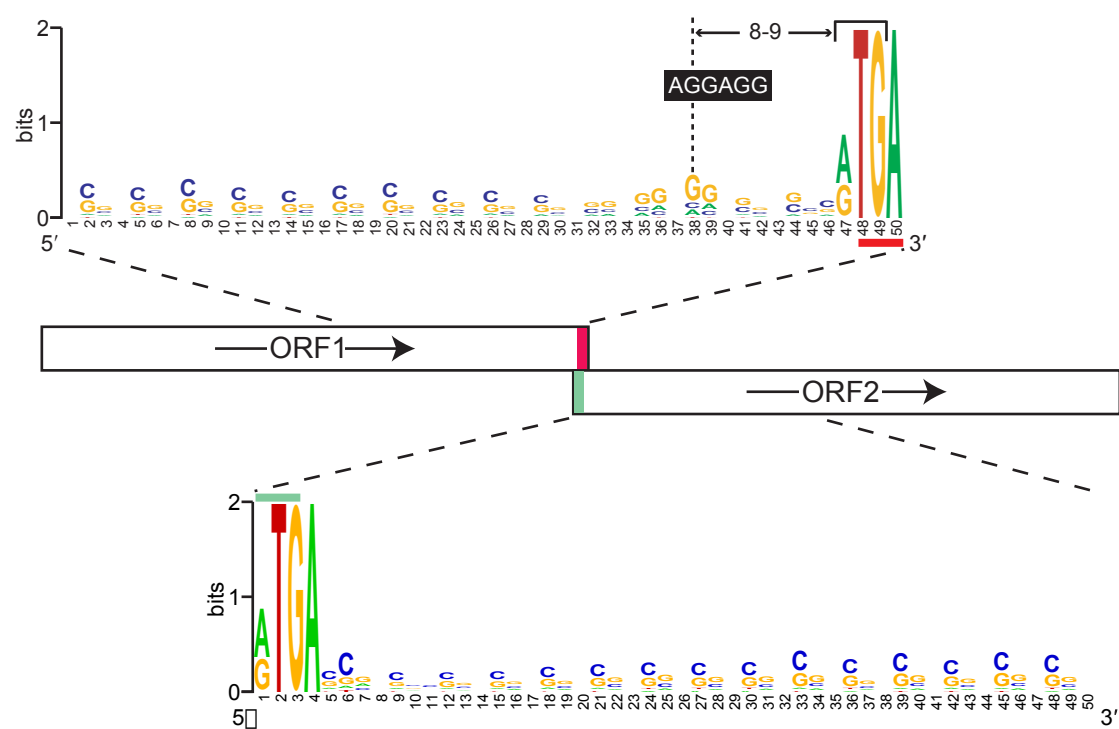

Supplement: S6 Fig — Both upstream and downstream ORFs showed the expected GC codon bias. The wobble positions of the upstream gene reflect the Shine-Dalgarno consensus sequence (black box), suggesting the coexistence of coupling and canonical translation initiation at these junctions. (PDF) [file pgen.1005641.s006.pdf]

Supporting Information Figure S7

A

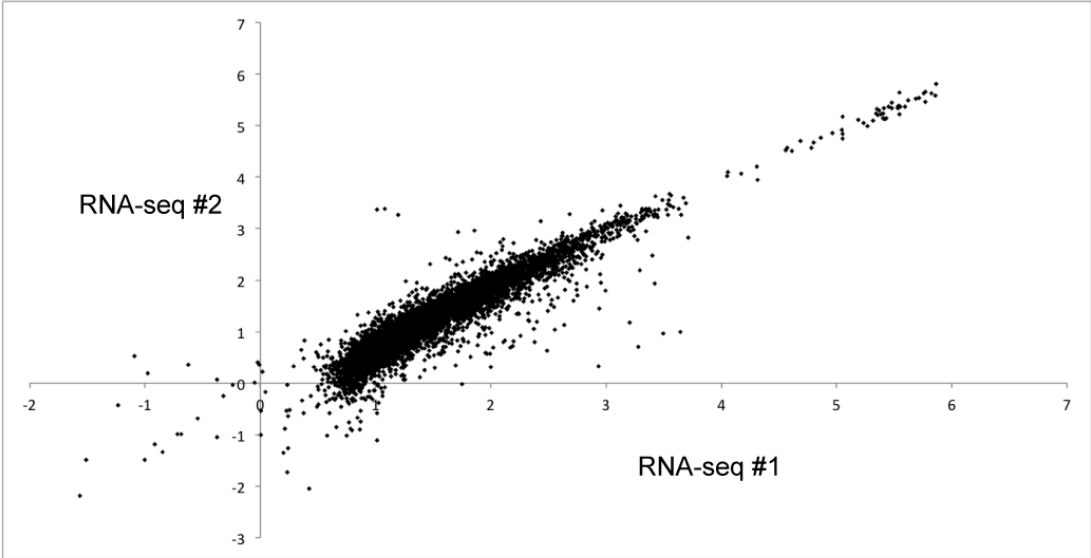

B

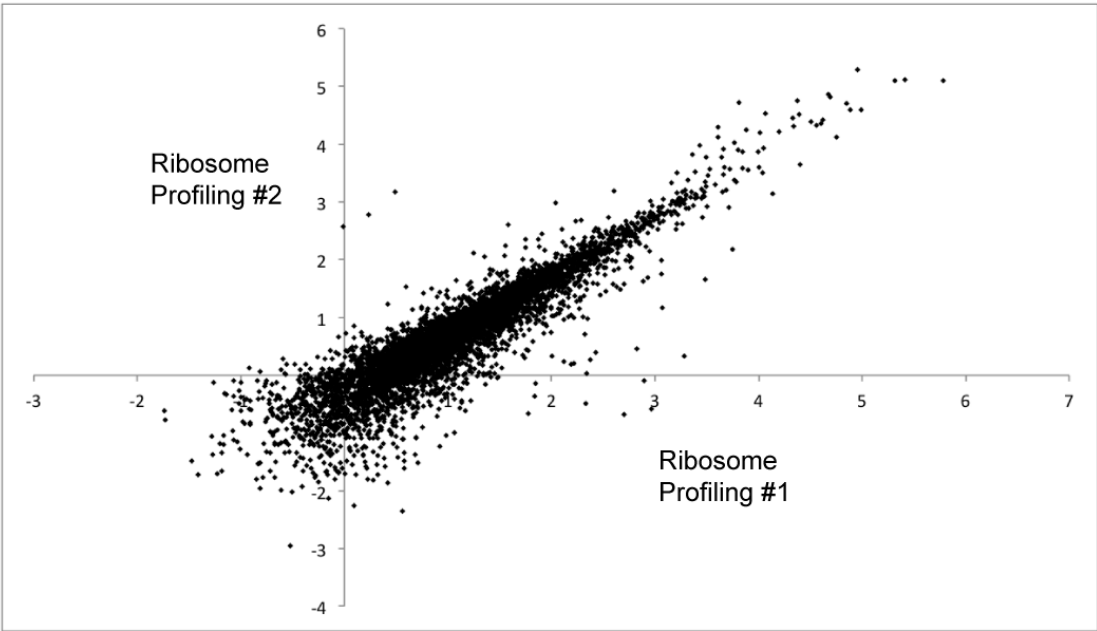

Supplement: S7 Fig — M. smegmatis transcriptome datasets were generated in duplicate for both the RNA-seq and ribosome profiling. Compiled read counts from genes in each dataset plotted on log axes show the highly correlative line of identity typical of genome scale studies. Read counts for a given gene in each experiment were normalized for gene length (average reads per nucleotide mapped along the gene), converted to log scale, and plotted as shown. Poorly expressed genes averaging a read depth of less than 1 have negative log conversions. RNA-seq (A) and ribosome profiling (B) replicates are shown. (PDF) [file pgen.1005641.s007.pdf]
